# Clinical Review and Hospital Reports

**Published:** 1834-10-01

**Authors:** 


### Clinical Review.

### St. George's Hospital.

### I. Clinical Remarks on Ununited Fractures. By Mr. Brodie.*

A case of ununited fracture which had been received into the hospital, gave rise to some clinical remarks, by Mr. Brodie, on the methods which have been adopted for the cure of this unpleasant accident.

Mr. Brodie considered, in succession, the employment of the seton—of pressure—the removal of the fractured extremities of the bone—irritation of those extremities, and the subsequent retention of lint between them. We may mention, before we proceed to particulars, that when a fracture is not repaired by bony union, it either unites by the intervention of a ligamentous substance, or a false joint is formed between the ends of the broken bone. When the latter is the case, those ends are covered with a ligamentous structure, and surrounded by a capsular ligament lined with a synovial membrane. In the case which formed the peg upon which the clinical lecture was hung, the former condition obtained.

In alluding to the employment of the seton, Mr. Brodie observed that Dr. Physic tried it in three cases, in two of which it was attended with success. Experience would appear to shew, that it rarely succeeds in the lower extremity.

"Amongst the cases in which Dr. Physic adopted it, I have been informed that there was not one case of ununited fracture of the lower extremity in which it did not fail: indeed, I am not aware that there is a single case on record in which it has succeeded in the lower limb, excepting one which was under my care. The patient was a boy, in this hospital, with an ununited fracture of the thigh. A seton was introduced between the broken ends of the bone, and he recovered."

Mr. Brodie alludes, in a favourable manner, to the mode of pressure employed by Mr. Amesbury. Mr. Brodie has known it prove successful—indeed, it answered in a case of Mr. Brodie's which occurred in the hospital last Autumn.

"In former times, it was a common practice to cut down upon the bones engaged in an ununited fracture, and to remove their extremities with a saw: in other words, to make a severe compound fracture. This practice has been, I believe, occasionally successful; but the instances of its success have been very few, compared with those of its failure. I remember conversing with an old surgeon of eminence, who had had frequent opportunities of seeing this practice resorted to, and he told me that he had not himself known a single case in which it had been attended with advantage. But, at any rate, there would be a decided objection to it in this case; for if I were to remove much of the tibia with the saw, the severed ends would not come in contact again, unless I were to perform the same operation on the fibula; and this would be altogether a frightful operation, attended with considerable danger to the patient. What, then, can be done? Sir E. Home was accustomed to mention, in his lectures, delivered in this hospital, a remarkable case which was under the care of Mr. Hunter. A patient had an ununited fracture of the humerus, in which a false joint had formed. Mr. Hunter cut down upon the part, and having introduced a spatula, scraped the fractured ends of the bone; inflammation followed; lymph was effused, and became organized. Bone was deposited in its centre; and in a short time bony anchylosis took place."

We may now glance briefly at the case which gave rise to the preceding observations. It was that of a little

* Med. Gaz. July 26th, 1834.

boy, about five years of age, who had broken both bones of the right leg, near its centre, two years prior to his admission into St. George's. Bony union had not been obtained, and continued pressure, under, we believe, the superintendance of Mr. Amesbury, as well as the introduction of the seton, had been tried without effect. Under these circumstances, Mr. Brodie cut down upon the tibia at the seat of fracture, removed a ligamentous sort of substance that connected the extremities of bone, scraped the latter, and dressed in the wound with lint. He did not attempt a similar operation on the fibula, which was broken a little lower than the tibia, because it would be difficult, dangerous, and unnecessary.

II. CLINICAL REMARKS ON SOME CASES OF DISEASE OF THE URINARY ORGANS. By MR. CÆSAR HAWKINS.*

Mr. Hawkins has communicated to the public some interesting cases, and some valuable observations on diseases of the urinary organs. We shall take the opportunity of noticing three instances of disease of the kidney, in connexion with stricture of the urethra. We do this in order to illustrate the pathology of that serious disease, and to display the changes in various portions of the urinary apparatus, that follow an obstruction to the passage of urine through the urethra.

CASE 1.—*Stricture—Thickening of the Bladder—Chronic Inflammation of the Kidney.*

John Weighell, æt. 49, was admitted into St. George's Hospital in August, 1833.

He had laboured under a stricture of the urethra for twenty years. When admitted, the smallest catgut bougie could not be passed. There was a hard cartilaginous tumor, inclosing a cavity in the perinæum. The urine was abundant, pale, highly alkaline; it contained a peculiar mucus, resembling a powdery matter, which slightly floated in it, and was intermediate in appearance between mucus and albumen. On applying heat, or adding nitric acid to the urine, its coagulation proved the presence of albumen. There was slight occasional pain in the loins, which, on several occasions, was mitigated or temporarily relieved by a blister.

Instruments were gradually passed through the stricture, but not into the bladder, their point being obstructed by what appeared to be an abscess in the prostate gland. The latter conjecture was supported by the circumstance, that a good deal of pus was evacuated separately from, and before the urine, the passage of which it occasionally obstructed. The progress was so far calculated to give encouragement, when a train of symptoms was established, which Mr. Hawkins has described with vigour and correctness. We shall venture to extract the description, and the commentary added by the lecturer.

"You have seen Weighell very nearly dying of a sudden increase of renal disease, to a return of which he is still liable at any time. It was in January last that he became low-spirited and out of health; then he had occasional rigors, pain in the back and groins, with more difficulty in making water; then in a few days he had a great quantity of blood in the water, which came suddenly, and continued for two or three days; and before it ceased, there came away with the urine a very large quantity of pus, which continued for some little time, and then ceased. While he was at the worst, he lay in a state of listless half stupor, with a quick, feeble, intermitting pulse, and the brown tongue of typhus fever; and he had occasional delirium, and was frequently crying from extreme depression of mind, when not asleep and stupid. These symptoms continued, more or less, for nearly a month, when he began to revive and recover strength; and the symptoms I have mentioned, both local and constitutional, and as connected with the urine, gradually went away, and left him as you now see him.

* Med. Gazette for June 21st and July 26th.

Now, what I believe to have happened during this time, was the formation and bursting of an abscess in one kidney, and that probably the right, from an affection of respiration, with pain on that side, which he laboured under for a few days. The discharge of pus from the kidney occurs in three different states—first a quantity is secreted from the tubular structure of the kidney, and from the infundibula and pelvis, without any cavity like that of an abscess, and while the cortical substance is only inflamed. I have seen this discharge take place suddenly, and to the amount of many ounces daily; so that it seemed almost impossible that it could have happened from the secretion from a mucous surface only; and yet dissection has shewn that it did so. Secondly, you find small quantities of pus partially confined in cysts, consisting of the infundibula and tubes, enlarged and dilated; these cysts communicating with the excretory tubes. Thirdly, you find circumscribed abscesses in the kidney, not communicating with the excretory tubes; even these, however, you can often trace to the commencement of the tubes, where a drop or two of pus, confined by adhesive inflammation, become the origin of larger collections of matter.

I judge that Weighell had a circumscribed abscess, from the rigors, and so on, *preceding* the purulent discharge, and because there was also a large quantity of blood before the pus, as if the wall of an abscess had been ruptured to give exit to its contents. If I am right on this point, the abscess has since probably filled up."

The treatment employed during this condition of stupor were stimulants, of course—a blister to the right side—and a blister, also, to the nape of the neck.

The sequel of the case may be briefly stated. The patient became gradually reduced in strength—emaciation made progress—about the 20th of June he was attacked with diarrhœa, attended with much general pain in the abdomen—and he died on the 6th of July, having suffered for a day or two from pain in the head, unaccompanied, however, with actual stupor. During the period that elapsed between the comatose attack and his death, the urethral symptoms continued nearly stationary. The suppuration from the abscess that was thought to exist in the prostate was diminished, and the cartilaginous tumor in the perinæum had increased. A catheter was occasionally passed.

*Dissection.* The cartilaginous tumor in the perinæum was placed anterior to the stricture, and contained a small abscess, communicating with the urethra. The stricture itself was broad and firm, white in appearance, and situated rather on the left side of the urethra. A catheter of tolerable size could be passed through it. The prostate gland contained an abscess, capable of holding a table-spoonful of pus. At the side of the verumontanum was an opening into the abscess, in which the extremity of the catheter had frequently been involved. The bladder was thickened to the extent of more than half an inch—its muscular fibres were prominent and enlarged, and its mucous membrane was dark-coloured, vascular, and folded into numerous little pouches. The ureters, the pelves of the kidneys, especially the left, and the infundibula, were in some degree enlarged, and their mucous membrane was inflamed. The secreting structure of both kidneys was condensed, and of a yellow colour; the left, which was nearly of its natural size, was more vascular and brittle than the right, which was diminished to half its natural bulk.

The chain of consequences resulting from the stricture of the urethra, and the lodgement of urine in the bladder, in the ureters, and in the pelves of the kidneys, are obvious even to the inexperienced pathologist. Perhaps it may be briefly, though not quite correctly, summed in the expression, that chronic inflammation was induced in all the portions of the urinary apparatus affected by the urinary accumulation. The case is interesting, because it informs us what we may expect, and what we should avoid. Mr. Hawkins judiciously dwells upon the circumstance, that pus may be secreted from the mucous membrane of the kidney, in a manner and to a degree which might reasonably warrant the suspicion of a circumscribed abscess in that organ.

CASE 2. — *Stricture — Effusion of Urine—Inflammation of the Kidney.*

Pollard Tate, æt. 33, admitted June 27th. He had suffered from stricture, with occasional retention of urine, for six years prior to his admission; but he had not adopted any treatment for the malady. Five days previous to admission, he felt, when in bed, a desire to make water; but merely a little dribbled away with much straining, and subsequently it was only voided in drops. On the following day, the penis began to swell, and, on the next, the swelling extended to the scrotum; on this day he had rigors.

On his admission, the penis and scrotum, the perineum, and lower part of the abbomen, were enormously swelled, and of a vivid red colour: he was in very great pain, with some fever, and the bladder was much distended with water.

"Now, I told you, some time ago, when speaking of effusion of urine, that I liked, if possible, to pass a catheter into the bladder, and in making the necessary incisions, to let one of them reach the instrument in the urethra, near the usual seat of the stricture. I found, however, here that the catheter would not at first pass, from the tense state of the parts, and from the quantity of sloughs in which the point was obstructed, even near the end of the penis; I therefore made several incisions in the scrotum and penis of considerable depth and length, and another in the perineum, down to the usual situation of the effusion; which last, in fact, gave exit subsequently to some urine, besides that which came through the catheter. I gave him fifty drops of laudanum, and had him placed in a warm bath as soon as the hæmorrhage ceased. Two hours afterwards he was much more comfortable; the swelling was not above half the size it had been previously; and after some little difficulty, the catheter now entered the bladder, and drew off the water which distended it."

The skin was prevented from sloughing by the incisions—sloughs of cellular tissue came away—and though erysipelas supervened, it was mild: and all appeared to augur favourably for the patient. We should state that, four or five days after his admission, a calculus, like a grain of coffee in appearance and in shape, was removed from one of the incisions in the penis. It seems not unlikely that this was the indirect consequence of the stricture, and the immediate cause of the effusion of urine —sticking in the narrowed passage, it might totally obstruct it.

On the 3d of July, an unfavourable change was noticed. The patient became affected with sudden and complete prostration of strength, a copious perspiration, and coldness of the hands and feet. Stimulants were of little or of no avail in removing these alarming symptoms. On the 6th, there was slight diarrhœa, and a little tenderness of the abdomen, and on the day on which he died, the 8th, pain was experienced in one wrist-joint.

*Dissection.* "The bladder was nearly in the same state as Weighell's, much thickened, and reticulated, and inflamed, and contained a good deal of thick bloody urine. The ureters were very much dilated and inflamed, and both of them were twisted and obstructed near the kidney, as you may see, by other preparations in the museum, is often the case in stricture. The pelves of the kidneys, and infundibula, were much more distended than in Weighell; in a high state of inflammation; and the kidneys themselves were coated with thick yellow lymph; and all these parts were on both sides full of a dark, bloody purulent secretion, quite pulpy in consistence, and which had passed down in great quantity into the bladder. The structure of both organs was inflamed, but not yet condensed or diminished in bulk, as in Weighell. In the right kidney, where most lymph had been deposited, one or two of the infundibula looked like distinct abscesses. Subsequent examination, however, shewed that this was not the case, the cavities all communicating with the pelvis of the kidney by continuous mucous surface."

Mr. Hawkins directs particular attention to three important circumstances:—the absence of distinct symptoms characteristic of renal disease—the abundant secretion of purulent matter from the mucous membrane of the kidney and ureter—the sudden supervention of symptoms of prostration, and the speedy occurrence of a fatal termination. The first should teach us caution—the second should enlarge and correct our opinions—and the third may diminish our surprise at the comparatively sudden manner in which some patients die after the operation of lithotomy, or during the progress of a case of stricture, when the local disease appears to be yielding in a satisfactory manner.

### III. Report on Hernia, consisting of Cases extracted from the Note-Book of Mr. Cæsar Hawkins; with Clinical Remarks.

There are few subjects which on the whole present such a variety of interesting points for consideration, and in which the result of the surgeon's reflexions are of more serious importance than the treatment of hernia; for ourselves at least we can safely assert that we scarcely ever saw a case in which we did not learn something, or in which some interesting or novel point did not occur to strengthen or modify the opinions we had seriously formed. The most important point however in the whole subject is the conviction on our minds of the propriety of operating at an early period of strangulation; often have we seen a patient sink under the disease for want of an operation, either from the ignorance or timidity of the practitioner, but it is very seldom indeed that we have witnessed the death of the patient purely from the operation having been performed. Even in the practice of hospitals we can ourselves recollect a complete change in the treatment of these cases, most of which when presented at the hospital are already of considerable standing, and yet when we began our studies we can recollect repeated consultations being held upon them after admission, before recourse was had to an operation; now on the contrary, if fair trials had been made beforehand, or if the strangulation has already lasted some time, no time is lost in what are then most probably only fruitless attempts at reduction, but the surgeon at once proceeds to the certain relief afforded by the operation; and nothing can be more striking than the comparative result of such cases. How little the system is affected by the operation when performed soon, the following case will shew.

1. *Strangulated Hernia—operation early.*

Charlotte Kitter, æt. 34, admitted Feb. 19th, 1834. Catamenia present. Has had femoral hernia for some years, reducible till 10 o'clock, a. m., after which attempts were made to reduce it without success, and in the evening she was sent to the hospital. There was a femoral hernia of some size, turning over Poupart's ligament, very hard and firm at the ring, and the tumor itself tender and painful. The chief circumstance Mr. Hawkins said, in his opinion, to be attended to in strangulated hernia, is the state of that part of the protruded bowel or omentum which is *embraced by the sac,* for if that portion is very hard and tender, it is not very likely to be reduced, and the part below may be quite flaccid and unattended with pain, although mortification may be going on; a portion of tightly strangulated bowel being defended by omentum or by a quantity of fluid in the sac below the ring. For this reason Mr. H. thought it right in this case to proceed at once to the operation, without further trials to reduce it, which was done about twelve hours after the strangulation. The sac contained about five inches of intestine, which was dark coloured, but quickly recovered its proper circulation when the stricture was divided, which was very broad and tight. After the bowel was returned a large quantity of serous fluid came down from the abdomen. She was then left quiet for the night.

20th. An injection was given, and afterwards three doses of Epsom salts and infusion of roses, and the bowels still continuing confined, three grains of calomel, followed by some castor oil, were also given. In the evening the bowels had been freely open, and no pain whatever was felt.

22d. Beef-tea was given, and the next day fish, the patient not having had a single bad symptom. On dressing the wound it was found to have almost united by the first intention. A small portion of skin, however, near the incision, looked as if it was going to slough from the force used in the taxis, which did actually take place. The patient however quickly got well.

2. *Incarcerated Omental Hernia—no operation.*

But while strangulated hernia cannot be operated on too soon, there are frequently occasions for the exercise of judgment on the part of the surgeon in determining whether strangulation is actually present or not. An old and large intestinal hernia for instance frequently causes attacks of severe pain, constipation, tenderness of the tumor, sickness and vomiting, and yet no operation may be necessary in such case, the bowel being merely *incarcerated,* i.e. its functions impaired by its confinement, (generally from some imprudence in diet,) and yet the stricture not being tight enough to endanger the circulation in the protruded viscera; in which case the symptoms may be combated by appropriate means, and the temporary obstruction overcome. It is in cases of this description too that the operation becomes really dangerous, from the size of the tumor, and from the adhesion generally formed, and hence the propriety of that line of practice recently again recommended by Mr. Key, though so often lost sight of, viz. when strangulation actually takes place, to divide the stricture without opening the sac. With omental hernia again, although an operation is sometimes as urgently required as for intestinal hernia, yet there is no doubt that as a general rule more room is afforded for the trial of such means as may succeed in removing the effects of the stricture without an operation. There is danger, indeed, of a portion of bowel being concealed in a mass of omentum, so that if from the *symptoms* there appears reason to fear such an occurrence, the surgeon should not trust to the feeling only of the tumor, but rather operate unnecessarily than incur the risk arising from delay; if, on the other hand, the symptoms are mild, he may wait till more urgent necessity for operation arises, and so perhaps avoid it altogether.

Ann Tenison was admitted under the care of Mr. Hawkins, Nov. 15th, 1833, with femoral hernia. She had had rupture for six months, and the present tumor has continued down for the last fortnight, producing no pain or inconvenience till four days ago, when symptoms like the present came on, and after a few hours suffering again left her; they returned, however, a second time, last night. The tumor is not very large, it is moveable and circumscribed, somewhat elastic, and has a large gland over it; it receives a slight impulse on coughing; is very tender, and painful even when not handled,—she has also a good deal of pain in the back and around the umbilicus. The bowels have been open since the tumor has been down, and she has no sickness;—tongue white—pulse not very quick.

On her first admission she was placed in a warm bath, and had 20 ozs. of blood taken from the arm, and an unsuccessful attempt was made to reduce the tumor. Mr. Hawkins saw her in the middle of the day, and thought that the tumor was omental only—that the stricture was not very tight, from a slight impulse being still perceptible on coughing—that it was incarcerated, and not strangulated, and consequently that an operation would probably not be necessary; in which opinion the other surgeons who were in the hospital concurred.

A dozen leeches were applied over the tumor, an injection administered, and some aperient medicines given by the mouth. By this treatment the pain was reduced, and the bowels acted upon several times; she slept well during the night, and had no return of symptoms, and in a few days she was ordered to wear a truss upon the tumor, with a not very forcible spring, and the pad not so convex as usual.

3. *Wearing a Truss upon an irreducible Hernia.*

In speaking of the propriety of wearing a truss over the omentum in the case just related. Mr. Hawkins said that if care was taken not to use too great pressure, nor at first to continue it for too long a time at once, there was no danger of producing inflammation or other mischief in the part, and that the truss served effectually to prevent further protrusion, even if it did not succeed in reducing the hernia altogether. He then related the following case to shew that the same treatment was also applicable to intestinal hernia, and being an interesting one, we preserved notes of the account. A young gentleman, about 13 years of age, was under the care of a physician for some time, in consequence of great disturbance of the bowels, and consequent derangement of the whole system, the cause of which was not evident till the boy complained of pain on one side of the abdomen, when it was clear that the testes had not yet reached the scrotum, and the pain was found to be just above the ring on one side. I was then asked to see him, and ascertained that without having come through the external ring, there was an effort to protrude on the least coughing or other exertion, the bowel apparently filling the inguinal canal on both sides. I then directed a double truss to be worn not pressing upon the external ring, where the testis would have been compressed, but having a broad pad bearing upon the course of the inguinal canal. The boy immediately improved in health, and after wearing the truss for about a year, the testes had reached the scrotum, and the incipient double inguinal hernia was completely cured. Something of the same kind as this Mr. Hawkins said was very common about the time of puberty, but the same boy was about a year afterwards an example of a circumstance which is comparatively very rare. He was observed to have very great irregularity in the action of the bowels, which were sometimes obstinately constipated for several days together, resisting the strongest purgatives, and only yielding at last to repeated doses of castor oil; the attack of constipation being frequently succeeded by diarrhœa for some time, during which time the evacuations frequently shewed a good deal of blood. Being under the care of the same physician, he frequently examined the abdomen, and I also saw him with the same object, but it was evident that there was nothing now wrong in the inguinal canals, and no complaint was made which led us to suppose that any other hernia existed to account for the severe symptoms he occasionally laboured under, and which had a good deal affected his health. At last, however, he fortunately received an accidental kick in the groin from a child, which drew attention to this part, and then a very small body was felt quite deep under Poupart's ligament, which was soft and slightly tender, and in which it seemed that our patient had occasionally heard something like the noise of air. This immediately excited our attention, as perhaps a femoral hernia, although, if so, it must be a very small portion of one side only of the bowel, not including the whole calibre of the canal, and scarcely equalling a common gland in size, and therefore, as it seemed, scarcely sufficient to account for the symptoms he had laboured under for several weeks, as no pain whatever had been felt in this part. Mr. Brodie also saw him with me, and said that he had never seen anything of the kind before. We agreed, however, that he should wear a slight truss over the part for some time. The effect of this was quite surprising, for the action of the bowels directly became regular and the health better; in a short time, nothing could be felt in the part, and he soon after left off the truss without having since had any return of similar symptoms.

4. *Rupture of Intestine by a Blow upon a Hernia.*

The records of forensic medicine possess more than one such case as the following, and shew the danger of leaving a hernia unreduced, and unprotected by a truss, however common such carelessness unfortunately is in all classes of life.

Henry French, æt. 55, admitted Jan. 11th 1834, under the care of Mr. Hawkins. This man had been leading a horse drawing a cart, when the animal took fright, and ran against some iron railings, by which means he was squeezed between the railings and the wheel. He had slight scalp-wounds in one or two places, and a severe contusion of one arm; but what he chiefly complained of was severe pain in the abdomen, especially on the right side, where there was an inguinal hernia, that he thinks was down at the time of the injury, and which came down also several times after his admission. There was no great anxiety or collapse when first seen, the pulse was 84, full and soft. Leeches were applied to the part, and saline and antimonial medicine ordered. The next day he seemed pretty comfortable, and said he was relieved by the leeches, though there was still some tenderness. Leeches repeated, with aperient medicine, as the bowels were not open. Pulse 90 and small. In the evening he felt better, though some distension of the bowels was perceived. Between 12 and 1 o'clock the house-surgeon was called up to him, and found him in very great pain over the whole abdomen, with exquisite tenderness to the least pressure—constant retching, much distention of the abdomen—the skin cold, and the pulse hardly perceptible;—in short, the most acute peritonitis had evidently come on. He continued much in the same state till about three, when he aroused himself, and asked for some warm gruel to drink, but before the nurse could procure it he had fallen back, and expired about forty hours after the injury.

On examination the next day, nothing was found in the hernial sac, but in the right iliac fossa a portion of small intestine was seen in a cavity formed by adhesion from recent lymph, and inclosing a quantity of lymph and pus and a little fæcal matter;—the bowel was slightly ecchymosed, and a small rupture of its coats was seen about one-third of an inch in length. The whole peritoneum was inflamed and coated with lymph.

The hernial sac presented a curious complication, that in an operation might have caused some difficulty. Below the sac was a hydrocele, and behind the sac a large varicocele, and in front of it was a fatty tumor about 2½ inches long, and surrounded by a smooth cyst, in which it lay loose like a portion of omentum in a hernial sac; so much so indeed, that it was thought at first there must have been a double hernia.

5. *Hernia produced by a Blow.*

William Cooper, æt. 20, admitted July 29th, 1834, under Mr. Hawkins, with the following history. He said that a fortnight before, a person had kicked him on the groin and scrotum, and driven the testicle up into the abdominal ring, whence it did not return for an hour, and that then a rupture had come down with it; that the bowel was supposed by his medical attendant to have been slightly strangulated at first, as it did not go up again for two days, during which time he had a good deal of pain, and complete constipation, and that he had had much pain in the abdomen since that time. There had been no swelling whatever of the testis, so that a mistake as to the nature of the case could not have been made as to this point, and he said that whenever he walked since the accident the rupture had come down with some pain, and receded again on his lying down. On his admission there was a good deal of tenderness of the abdomen, especially on the right side, but without any hernial protrusion. This pain was increased by exertion, especially when he made water, which he did with more difficulty than usual—the spermatic cord seemed full and tender, but the testis was healthy. He had also some degree of fever.

A calomel purgative with castor-oil was given, and some leeches applied to the abdomen, by which he was somewhat relieved.

Sept. 1. Some pain complained of chiefly in the epigastrium—feverishness lessened.—Purgative and leeches repeated.

2d. Not able to make water at all, and the catheter drew off a small quantity of dark coloured water. Calomel purgative repeated.

3d. Not much pain—pulse returned to its natural state. Sickness and uneasiness in the epigastrium, with some bilious vomiting. Effervescing saline draughts. Calomel half a grain, and opium half a grain, to be taken night and morning.

7th. Some return of pain, and considerable nervous anxiety—no fever—leeches repeated.

8th. Pain all gone.

13th. No pain in any part. The hernia has not been down since his admission. Reported cured.

*Treatment after Operation for Hernia.*

There is much more room for surgical skill than is usually imagined after the operation for hernia, for it is by no means an universal rule that inflammation is the chief danger to be guarded against. No doubt the effects of the stricture or of the stricture and the operation together are very often to produce rapid and fatal inflammation of the peritoneum, but we are sure that we have seen some patients sink after the operation, in whom not a trace of inflammation has been present, and who might probably have been saved by a different line of practice to that which we saw adopted. We recollect some years ago two very instructive cases which we saw under Mr. Hawkins's care, an account of which will be found in the Medical Gazette, Vol. VI., p. 927, both of which were probably saved from the immediate effects of the strangulation of several days' duration by being well supported after the operation. These are less common perhaps than cases in which there is a mixture of chronic inflammation with constitutional debility or irritation. The different practice to be adopted according to the variation of symptoms in such cases is of material importance therefore; and as it is a point to which Mr. Hawkins often directs the attention of the pupils, we have selected the following cases to illustrate some of the circumstances occasionally met with.

6. *Operation followed by great Debility and Irritation.*

Susan Dixon, æt. 44, admitted Oct. 22d, 1833, under the care of Mr. Hawkins, with a femoral hernia of some size, turning upwards over Poupart's ligament. She states that she has suffered from a rupture for 13 years, during which time she has never worn a truss; she has never been without a swelling in the groin, which has increased after any exertion, and she has suffered from frequent "bilious attacks." A year ago the rupture was strangulated, though without such severe symptoms as at present, and at that time the tumor was larger than it now is. She was seized with the present attack while scouring the floor on Saturday morning the 19th, (three days and a half ago,) since which time pain in the bowels, vomiting, and excessive languor have continued almost without intermission till her reception in the hospital. During this time she was treated for inflammation of the bowels, with 40 leeches, purgative medicines, and a large blister!—the hernia not having been discovered till this morning. On her admission she complained of great tenderness on the tumor and in the abdomen, especially in the part immediately around the tumor;—there is very distressing and constant vomiting of dark brown substance, partly fæcal in smell;—the countenance sunk and sallow, and with an expression of great weakness and anxiety. Bowels not opened since Friday the 18th—tongue white and furred—pulse quick and weak.

The patient was kept in a warm-bath for a considerable time, but without a reduction being effected; Mr. Hawkins therefore proceeded to an operation.—The steps of the operation were remarkable for the quantity of fluid contained in the fascia propria, and its exact resemblance to a hernial sac, especially as the real sac was so exceedingly thin and transparent, that when the fascia was opened and the fluid let out, it looked like the immediate peritoneal investment of the bowel. So distinctly was this part seen through the sac that many spectators thought the operator was cutting into the bowel, especially as it was some little time before Mr. Hawkins could satisfy himself of the exact nature of the parts, a great number of serous bands at the reflexion of the fascia propria adding still farther to the obscurity, as they looked exactly like adhesions between the bowel and the hernial sac, for there was no distinction in appearance between the outer surface of the real sac, and the inner surface of the fascia propria, both being as smooth as any serous membrane. When the real sac was finally opened, three or four inches of bowel were seen, which was very dark, softened, and ecchymosed, especially where the two folds of intestine were embraced by the stricture; the bowel being pulled down after the division of the stricture to examine its appearance, which shewed very strikingly the changes produced by the stricture on the part below.

23d. The operation being performed in the evening, she was left quiet during the night, and in the morning an injection was administered, which brought away a good deal of fæces. The sickness was instantly removed by the operation, and had not returned. Pulse fuller but weak and slow, and intermitting every 20th beat.

Some beef-tea allowed.

℞. *Calomel*, gr. iij. *statim*.

*Mag. sulph.* ʒij. *Aquæ menth. pip.* ℥iss. *M. 4tis horis sum.*

24th. Streaks of blood in the evacuations procured by the medicine, which Mr. Hawkins said he had several times seen where the bowel was much injured by strangulation, and he recollected one case in which more than a pint at a time had thus come away, the patient however ultimately getting well. The pulse is more slow, and intermits much more frequently. The water requires occasionally to be drawn off by the catheter. Abdomen tender and full, as if from flatulence.

To take a little white wine, and some brandy occasionally in water and with arrow-root.

℞. *Sp. æther. nitros*, ʒj. *Mist camph.* ʒx. *M. 6tis horis.*

*Vespere.* Feels stronger and better, but complains much of flatulence, amounting almost to tympanitis. P. intermits less frequently.

Ordered a large poultice of chamomile flowels in hot water, which gave great relief to the abdomen.

25th. Bowels open, a little blood still coming away. Tympanitis continues, by which the abdomen is rendered of great size. Pain and tenderness however less. P. stronger and fuller, and not at all intermittent. Tongue clean. Countenance less anxious. Mr. Hawkins ordered her to *lie upon her side*, which assisted in some measure in expelling the wind. Some relief was also given by the introduction of a *large catheter* into the rectum, through which some wind came away, which the sphincter otherwise retained. Most relief, however, was subsequently given by binding a broad *flannel bandage* pretty tightly round the abdomen. The wound dressed and looking healthy, though suppurating in part.

℞. *Haust. salin. ammoniat.* ℥iss.—*Træ. opii*, ♏v. *M. 6tis horis.*

27th. Better—bowels open—pulse stronger—tongue clean.

28th. Sickness last night, otherwise much the same. Tympanitis a little less. Bowels not open, and some solid fæces felt in the colon.

℞. *Ol. ricini*, ℥j.
*Rep. pil. calomel, hss.*

30th. Still a good deal of pain in the abdomen occasionally, to which a *mustard poultice* is frequently applied with advantage for a few minutes at a time. Wound opened again partly in consequence of suppuration.

Nov. 5th. She has gradually improved, and feels nearly as well as before the strangulation, being still however very weak and languid.

She has continued her wine and brandy, and as much food as she can take.

℞. *Infus. cascar.* ℥iss. *Acid sulph. dil.* ♏vj. *Tr. gent. c.* ʒss. *M. ter die.*

A sinus afterwards formed to some distance from the wound, which required to be laid open, but on the 28th she went out cured.

7. *Operation twice performed—Mortification of Bowel—severe Symptoms by Separation of Slough. ?*

James Wheatley, æt. 46, admitted July 21st, 1831, with strangulated hernia, for which he had worn a truss since he had been operated on by Mr. Hawkins for the same hernia, about a year ago; but he had allowed the rupture to come down occasionally, and it had not gone up again since Monday (three days ago), when, after some exertion, following a hearty meal of potatoes, a large portion of bowel had descended. Vomiting came on two hours afterwards, and had continued till the present time, and hiccough yesterday.

He complains now of violent griping pains in the abdomen—constant retching and vomiting of stercoraceous matter, with occasional hiccough. Abdomen tender around the hernia, and still more in the epigastrium, which is the chief seat of his pain—no motion since Monday—countenance anxious—pulse 76.

He at first refused to have the operation performed, but submitted in the afternoon, after ice had been applied for some time. Mr. Hawkins performed the operation with some care, in consequence of the former operation, and found the intestine in contact with the fascia, the peritoneal sac having been obliterated in front by the previous incision, so that, in the lower part of the sac, the bowel was closely adherent to the common integument. The intestine consisted of about five inches of the smaller bowel—it was of a bright red colour, from inflammation, and thickly coated with recent lymph, and, upon separating the adhesions, a spot, of the size of a shilling, was seen to be quite black, looking like coagulated blood, and without any glossiness, and the coats of the bowel very thin. After a little consideration, Mr. Hawkins returned it into the abdomen observing that the mortification of so small a portion could often be effected within the abdomen, so that the slough separated into the canal, and a better line was presented than if the surgeon was to open the bowel, and leave an artificial anus; and that, if the requisite adhesions did not take place for this object, still it was generally found that the mortified spot remained at the internal ring, and, consequently, that the fæces did not necessarily become extravasated into the abdomen, but could very often come away by the wound; the case demanding great attention, however, about the period when the slough would probably separate.

The vomiting, and hiccough, and pain continued after the operation; and, about four hours afterwards, 20 leeches were applied (fomentations not appearing to give any relief), and the following mixture—

℞. *Magn. sulph.* ʒjss. *Acid. sulph. dil.* ♏v. *Aq. menth. viridis,* ℥jss. *M. ft. haust. 2dis horis sumend.*

26th, 8, a.m. Tongue more dry—pulse 86, weaker—bowels just opened twice, the evacuations being very dark and fetid.

1, p.m. All pain and tenderness gone—hiccough continues—pulse very low.

Allowed beef-tea and arrow-root in small quantities. Ordered to continue the draught, and, in the evening, to have an injection of gruel, and to take some pills, with *Calomel*, gr. iij. and *Pil. sapon.* c̄ *Opio*, gr. v.

23d. More comfortable. Pulse a little stronger—bowels not at all free.

*Contr. Haust.*

24th. Tongue clean and moist—no pain or tenderness—complains only of hunger, but Mr. Hawkins allowed no solid food.

25th. Wound dressed, and found almost wholly united by the first intention.

27th. Some heat of skin—bowels very little open. Complains of griping pain.

*Appl. fotus papav. Contr. haust.*

29th (8th day.) Quite comfortable till this morning, about 1 o'clock, when he was attacked suddenly with violent pain and tenderness in the abdomen—constant vomiting, which soon became stercoraceous—tongue dry—pulse very small and rapid—great flatulence complained of.

An injection was given, which brought away no fæces—his mixture was repeated every two hours, and the abdomen fomented, without relief.

10, a.m. Symptoms increasing.

*Appl. Hirud.* xxv. *abdomini.*

1, p.m. Mr. Hawkins saw him, and found the symptoms not relieved by the leeches. Abdomen, quite tympanitic—very anxious.

*V.S. ad* ℥xij.

℞. *Calomel,* gr. v. *Opii,* gr. j. *M. statim.*

℞. *Aquæ menth. pip.* ℥jss. *Sodæ tartrat.* ʒiij. *Magnesiæ,* ʒss. *M.* 2*dis horis donec alvus respond.*

6, p.m. *Calomel,* gr. v.

10, p.m. Pain lessened considerably—has just had a copious motion—the retching and hiccough continue—pulse small and fluttering—extremities cold—surface of body bathed in cold perspiration.

*Appl. Empl. lyttæ magn. abdom.*

℞. *Calomel,* gr. iij. *Opii,* gr. ij. *stat.*

30th. Has a good deal rallied. The extremities began to get warm soon after the application of the blister. Skin now moist and warm—vomiting—pain and tenderness ceased—bowels discharging chiefly secretions without fæces.

*Contr. Haust. aper. salin.*

*Repr. Pil. vesp.*

31st. *Repr. Pil. et Haust.*

August 2d. Has very much improved, but has had several returns of vomiting and pain to a slight extent—bowels not free.

℞. *Pil. hydrarg.* gr. iv. *Pil. sapon.* c̄ *Opio,* gr. iij. *Extr. coloc. comp.* gr. v. *M. o. n. s.*

℞. *Olei ricini,* ℥ss. *mane sequente.*

*Beef-tea, &c.*

5th. Was again attacked this morning with vomiting and griping, without pain.

℞. *Calomel,* gr. iij. *P. opii,* gr. j. *M. statim.*

*Enema terebinth.*

1, p.m. Vomits *stercoraceous* matter—tongue dry—hiccough—not much pain.

*Repr. Pil. Cal. et Opii, statim et h. s.*

*Vespere.* Symptoms continue—extremities cold.

*Vini albi.* ℥ij. *Calomel,* gr. iij. c̄ *P. scamm.* gr. ij. *statim.*

*Olei ricini,* ℥ss. *mane.*

6th. Vomiting continues. Evacuations not fæcal for some time—surface still cold.

*Vini,* ℥iv. *Ova.*

*Empl. lyttæ abdominis, et postea Ung. hydrarg.*

7th. Beginning to rally a little—vomiting continues. *Burnt brandy,* ℥iv.

9th. Going on well. Continues his wine and brandy, and allowed also some porter.

13th. Going on well till to-day, when some griping returned, with vomiting, and again on the 20th, at which time the abdomen was very much swollen and tympanitic.

It is unnecessary, however, to detail the rest of the case, as it went on nearly in the same way for some time longer, before he was finally cured, during which time the treatment of the case was nearly the same—supporting the general strength, while the action of the bowels was restored by calomel and opium, and purgatives, &c.

Mr. Hawkins attributed the symptoms to the separation of a slough from the bowel, by which inflammation was produced, with a great deal of irritation and disturbance of the functions of the bowels, from the consequent narrowing of the canal at that part.

---

### LONDON DISPENSARY.

### BEST MODE OF TREATING MAMMARY ABSCESS.

Mr. Robinson has communicated six cases of mammary abscess to the Medical Gazette,* for the purpose of displaying its history and treatment. We presume that the greater number of our readers are tolerably familiar with the former—that few need be informed of the occurrence of common abscess in the breast, as in any other organ or tissue—and that it is not necessary to

* July 12, 1834.

describe at any length the mode in which obstruction of the lactiferous ducts may occasion inflammation, suppuration, ulceration of the integuments, and fistulæ.

The question which has been disputed in reference to the surgical treatment of this complaint, is the propriety or otherwise of an early opening. Mr. Robinson's observations on this head are not very copious nor precise. They are as follow.

"The third case proves, that where the abscess is owing to distention of the lactiferous tubes, the formation of fistulæ may sometimes be prevented.

The fourth case shews the result of opening by the lancet, and opening spontaneously. The former plan will generally be found the best, especially in deep-seated abscess. In this case the opening made by nature was large, and the contents of the abscess were evacuated all at once, which is more favourable than usual; for generally the opening is no larger than a pin's head, and is slow in healing up, the skin being commonly much destroyed before it gives way. Even in this case that abscess was much longer in opening, and more painful, which burst spontaneously, than that which was opened by the lancet.

The fifth case more forcibly proves that the artificial opening is preferable to the opening by nature; for the first abscess being opened by the lancet, was healed in three days; whereas that which broke of itself was much slower in healing, and was slightly fistulous.

This also points out the necessity of watching these cases: the woman was desired to call on me the following day, instead of which she allowed three days to elapse; otherwise I believe the fistulæ would have been altogether prevented.

The sixth case shews this disease in its most distressing and tedious form: the breasts were permitted to become over-distended with milk; both breasts were inflamed throughout; numerous abscesses, implicating the lactiferous tubes, followed; and she suffered severely for ten weeks.

The two last cases prove the great advantage of pressure (after the inflammatory stage is gone off,) in obliterating fistulous canals—a plan which will in many instances prevent, if it does not altogether supersede, the necessity of extensively laying open the breast, a custom which used to be resorted to formerly for the cure of sinuses, and which is even in the present day, I believe, occasionally adopted."

These remarks embody little more than the usual recommendations and the common practice. The most annoying circumstance connected with abscess of the mamma is the disposition to separate processes of suppuration, or, rather, to the formation and collection of matter in several portions of the same breast. They redound in some degree to the discredit of the surgeon, and form a serious source of distress to the patient.

It has been noticed in cases of diseased absorbent glands, and even in abscess of the cellular tissue, that suppuration does not always form in a single point, nor appear at the commencement inclosed in a single cavity. On the contrary, it has been found that it sometimes begins in several distinct portions of a gland, and that some time elapses before one common abscess is produced. The practical rule has been deduced, to wait till such a collection has occurred before an artificial opening is resorted to. If the surgeon operates too early, he has to open in succession several abscesses instead of one.

Our own opinion is, that the fact should always be remembered by the surgeon, and that by it his practice should be mainly guided. Yet we think that too much stress has been laid upon the one, and that the other has been carried rather too far. In cases of venereal bubo, if the surgeon declines to open the abscess until a large suppurating cavity is formed, he will probably endanger the vitality of the integument, and certainly protract to an unnecessary extent the duration of the complaint. Our readers will find more detailed observations on the management of suppurating bubo in another place.

We suspect that the cases of mammary abscess and suppuration in a strumous or venereal gland are not altogether analogous. In the instance of the gland its whole texture is diseased, and will probably be invaded throughout by suppuration. But the mamma is differently circumstanced. The lactiferous tubes are obstructed in one part — distention, inflammation, and suppuration in that part are the result,—and there appears to be no necessity why other portions of the gland should be involved. That they *are* so will probably be found to depend upon another cause. The gland is composed of numerous lobules, with adeps and cellular tissue interposed. Inflammation occurring in one part of the gland may readily extend along the cellular processes to another. The same thing would seem not unlikely to ensue, if matter were confined, or if it had not a free exit; and such is actually the case.

If these considerations are correct, an early opening of a mammary abscess is desirable. We are convinced, for our own parts, that it is so. We have treated many cases of this complaint, and were at first annoyed, as all surgeons have been, by repeated suppurations and ulterior sinuses. It appeared to us that these depended on a simple opening not sufficiently evacuating the abscess. After some practical experiments we adopted the following plan with much success.

Having satisfied ourselves that suppuration was actually established, we made with a lancet as dependent an opening as circumstances would admit, a vertical puncture being generally preferable to a horizontal one. We then introduced a probe into the cavity of the abscess, and ascertained if any sinus extended from it to another portion of the breast—or if the point of the probe could be made to project beneath the skin, in a more dependent spot than the puncture. If either was the case we cut on the extremity of the instrument, made its point project through the incision, tied around it a narrow piece of tape, or a silk ligature, withdrew the probe, and thus established a small seton between the opening originally made and that obtained by cutting on the probe, at the most dependent portion of the abscess. If more than one sinus extended from the latter, or if its formation or situation was such that one dependent opening was insufficient to empty it completely and at once, another incision was made upon the probe in the requisite direction, and another seton passed in the same manner as the former. The whole should be done at one operation, and since we have adopted this method of management, we have not been annoyed by repetitions of abscesses or formations of sinuses. We have found it in fact a great improvement in the treatment of this painful and troublesome affection. The operation is rather more severe at the time, but when once performed, it prevents the necessity in almost every instance for others. A patient will go through one with fortitude, but her spirits are broken and her strength of mind is shaken by its frequent repetition.

---

### DR. STOKES' LECTURES ON CEREBRAL DISEASES.*

*Neuroses—Encephalitis—partial Cerebritis—Phrenology.*

Dr. Stokes has made many judicious observations and reflexions on that mysterious class of diseases termed the neuroses. Those who look merely to affections of the brain and spinal marrow, as constituting the neuroses, take a very limited view of the subject. The nervous influence pervades every structure in the body, and deranges as well as presides over every function. In every disease, however structural or even mechanical, we may be assured that the nervous system of the part or the whole is affected.

" If we take, for instance, a case of gastritis, or hepatitis, we find a lesion of function in the nerves of the respec-

* London Med. and Surg. Journal Nos. 124, 125.

tive organs, which, in certain cases, seems local, but if the inflammation be intense and the fever high, we have superadded to this a sympathetic affection of the brain, or spinal cord. The same thing applies to all forms of local disease, for in all there is an affection of the nerves, either confined to the suffering organ, or extending to the whole system."

We find that affections of the cerebro-spinal system of nerves differ very materially from those of the ganglionic nerves. Most terrific and fatal diseases of the medulla spinalis may occur, without any disturbance of the mental functions; but these last seldom remain unaffected when the brain, especially its surfaces, becomes diseased.

"To follow up this point, suppose we take the diseases of the brain itself as compared with each other; we find that their symptoms vary according to the locality, so that whether we look to physiology or pathology we must consider the brain as consisting of several distinct parts, and not as an inseparable whole. It is admitted by many writers of high authority, that there is a difference between the symptoms of disease affecting the periphery, and disease affecting the central parts of the brain; and there is reason to believe, that we may be able in many cases to diagnosticate affections not only of the centre and periphery of the cerebrum, but even of other parts of the organ."

This is rational doctrine, and although it may be ridiculed by the antiphrenologists, as auscultation was by the anti-stethoscopists, we are convinced that the doctrine will gain ground as pathology advances.

Although there are several organic affections of the spinal marrow attended by pretty regular symptoms, and by which they may generally be recognized, yet, unfortunately for the practitioner and pathologist, there is a host of other nervous disorders, unconnected with any appreciable change of structure in the nerves or the nervous centres.—Epilepsy, tetanus, hydrophobia, chorea, and hundreds of other complaints, for which we have not even names, present themselves to the memory of the practitioner, and remind him that he has no insight into their pathology. They are lesions of function—the organic cause, if any, being unknown!

"No one can deny that neuroses are very different from organic diseases of parts. If we compare them with that class which is most familiar to us,—the inflammatory affections, we find a remarkable difference. In the first place, the neuroses may be brought on by causes not reckoned among those commonly capable of exciting inflammation. In the next place, their invasion is sudden, and their progress rapid; they arrive at their acmé in a very short period of time, and subside rapidly. These are characters which do not belong to the ordinary forms of organic disease. Again, we often observe the utmost intensity of nervous pain without the coexistence of swelling, redness, or heat of the part affected. We find, too, that they are not to be subdued by the antiphlogistic plan: on the contrary, several of them are either relieved or cured by an exactly opposite line of practice; and many cases which would appear to demand the lancet are known by long experience to be most benefited by stimulants. Lastly, the most accurate and well-conducted investigations of pathological anatomy have failed in demonstrating the slightest organic change in these cases,—at least, where changes are found, these are *neither constant, competent, nor commensurate with symptoms;* so that whether we compare the information we derive from symptoms, or the result of pathological anatomy, we find a great difference between neuroses and organic diseases. It may be said, that though they are not inflammatory affections, they have some resemblance to them. This, however, is only a gratuitous supposition; for even in the very worst cases they present nothing analogous to the results of inflammation, and the brain and spinal cord are as free from perceptible organic change in the majority of cases of fatal tetanus and hydrophobia, as they would be in nervous affections of a slight and transient character."

Dr. Stokes proceeds to the consideration of—1st, local inflammation of the brain—2d, general phlogosis—3d, mere sanguineous congestion—4th, apoplexy —5th, paralysis.

Our author believes that inflammation of the membranes of the brain may be sometimes distinguished from phlogosis of a portion of the cerebral substance; but when the whole substance of the brain is inflamed, our means of diagnosis fail. The distinction, however, is not of very material importance, in a therapeutical point of view. We pray attention to the following extract.

"If we inquire what are the symptoms of membranous inflammation of the brain, as laid down in books, we shall find them to be the following:—pain, delirium, convulsions, alteration of sensibility and coma. These are the symptoms which are generally given as characteristic of arachnitis; and it is quite true that they are observed in many cases of the kind. But the person must be dull indeed who thinks that such symptoms imply nothing more than an inflammatory affection of the membranes of the brain. Take for instance one of the most prominent symptoms—delirium; what does this imply? that the portion of the brain which discharges the functions of intelligence or mind has been injured, and is rendered incapable of performing its office. No one will venture to assert that the membranes of the brain are the organs of thought, and that the delirium proceeds from *their* morbid condition; such a notion as this could not be entertained for a moment. What then are we to suppose? One of these two things—either that there must be inflammation of the substance as well as of the membranes, or that the substance of the brain must be affected in a neurotic manner without any actual inflammation. As far as delirium is concerned, it appears to me to be quite impossible to distinguish between inflammation of the brain generally, and of its membranes. The same rule applies to the other symptoms, convulsions, alteration of sensibility, and coma. I repeat, that all we can say on this subject is, that in such cases there is either inflammation of the substance as well as the membranes of the brain, or that, with the membranous inflammation, there is a neurotic condition of the substance of the brain. Yet who, in such cases, can affirm with certainty that the symptoms of derangement of the substance of the brain are merely neurotic, when inflammation is admitted to exist within the cranium, and when we know that the two inflammations commonly co-exist?

The fact of delirium occurring so frequently in inflammation of the membranes of the brain, is of considerable importance, as showing, not that membranes of the brain have anything to do with intelligence, but as supporting the opinions of those who believe the periphery of the brain to be the seat of the intellectual faculties, and here is a fact which, as far as it goes, is in favour of the doctrines of phrenology. If we compare those cases of cerebral disease in which there is delirium, with those in which it does not occur, we shall find that it is most common in cases where disease attacks the periphery of the brain, as in arachnitis. The cases in which we observe great lesions of the brain without delirium, are generally cases of deep-seated inflammation of a local nature, or inflammation of those portions of the brain which the phrenologists consider not to be subservient to the production of mental phenomena. This fact, also, would seem to confirm the truth of the opinion of the difference in function between the medullary and cortical parts of the brain. It is supposed that the cortical part of the brain is the organ of intelligence, while the medullary portion performs a different function. It is, however, a curious fact, that in delirium the inflammation is generally confined to the surface of the brain, and that in cases of deep-seated inflammation, the most important symptoms are those which are derived from the sympathetic affections of the muscular system."

The foregoing remarks are very rational, and indicate the deep thinker, as well as the observant practitioner. When a portion of the brain, as in apoplectic effusion, tumors, or other morbid growths irritating the neighbouring parts, becomes inflamed, we find lesions of function in the muscular system, rather than in the intellect. This is often evinced, at first, by an apparent increase of innervation in certain muscles of the body—hence, pain in some of the muscles of the extremities is often an early symptom of local encephalitis. This is a curious fact, but one which appears to be well established.

"In partial encephalitis, there is often but little, or even no pain in the head, and the only warning we have of the approach of cerebral disease is the occurrence of pain in the extremities, followed by rigidity. Here are the two most prominent symptoms of the disease, pain in the muscles of the extremities, and then rigidity. Further, we have alternate spasms and relaxations of the muscles, in which, however, the power of the flexor muscles ultimately prevails, so that, if the disease be in the fore-arm, it may become permanently flexed on the arm, and the contraction of the fingers is sometimes so great as to drive the nails into the flesh. If it affects the leg, the heel may be pressed against the buttock sometimes so forcibly as to form a sore. As the case proceeds, the limb becomes more fixed in its new position, and every attempt to extend it causes pain. During the prevalence of these symptoms, it frequently happens that the patient does not feel pain in the head, or any diminution of intellectual power. The absence of pain in the part affected may be accounted for by recollecting that it is a general law that all inflammatory affections of deep-seated parts are, to a certain extent, of a comparatively painless character, and we may account for the non-existence of any lesion of the mind, by remembering that the disease is partial, and confined to a portion of the brain which appears to have little or no connexion with the intellectual functions. In cases of this kind, when the muscles of the face are affected, the phenomena are interesting, from their being *(in the first stage)* the reverse of those of apoplexy. The face is drawn *from* the affected side, and the tongue pushed, by the opposite half of the genio-hyo-glossus muscle, *to* the affected side. This is the spastic stage, when complete disorganization has not yet occurred. But when this happens, then the phenomena of the face are like those of apoplexy, because the opposite muscles, which were in a spasmodic, are now in a paralysed state, so that the face is drawn *to* the affected side, and the tongue pushed *from* it, by the healthy action of muscles which are deprived of their antagonists."

In partial or local encephalitis, delirium may not exist, because the part inflamed may not be employed in the exercise of the mental functions—or because the corresponding part in the other hemisphere may be unaffected.

The next stage of encephalitis is that in which the structure breaks down, as it were, and softens into a kind of matter. In this stage, a train of symptoms very different from the former set obtain. The rigidity and spasm of the muscles diminish, and are succeeded by a paralytic and flaccid state—voluntary motion in one side is lost. In the first stage (irritation or inflammation), a cure may be expected:—In the second, it is impossible.

"In the partial inflammation of the substance of the brain, sensation is variously altered. In some cases motion is lost, while sensation remains intact: in others, sensation is partially or wholly abolished. In many instances the intellectual powers remain in all their integrity, or but little impaired, even after the occurrence of symptoms which mark the softening down of the substance of the brain, and its conversion into purulent matter. In a few there is, during the first stage of the disease, a slight alteration in the state of the intellect, marked by a certain degree of excitement or exaltation of the mental faculties, and this, on the supervention of the second stage, is exchanged for a state of depression. In fact, the morbid phenomena of the mind and of the muscular system, where they co-exist, appear to be regulated by the same laws. Where the disease is extensive, you can easily observe the injury of the mental faculties which accompanies the second stage; the patient answers slowly when questioned; his memory is weak and his countenance has a stupid expression. But cases, even of extensive local suppuration, have been described by various authors, in which there was no lesion of the intellectual functions observed. These, however, generally admit of an explanation. Thus, in the cases recorded by Lallemand, the abscesses were situated in the cerebellum, pons Varolii, and other parts which are not supposed to have any connexion with the phenomena of mind. There are several well-authenticated cases of extensive disease, not only of these parts, but even of the substance of the hemispheres, occurring without any appreciable lesion of the intellect. Thus, Mr. O'Halloran gives the case of a man, who, after an injury which destroyed a large portion of the frontal bone, had extensive suppuration of the brain, and lost an enormous quantity of the substance of one of the hemispheres, and yet preserved his intellect entire up to the moment of his dissolution. There is some difficulty in explaining this. It is an opinion entertained by some physiologists, that, when one hemisphere is diseased, its functions are discharged by the other, and that the brain being a double organ, disease of one side does not impair the functions of the other. But in answer to this, it may be urged, that there are many cases on record, in which disease of *a single hemisphere* has produced great alterations of intellect. The supporters of the former opinion attempt to explain such cases in this way. They state, that in the majority of such cases there was, besides the local encephalitis, inflammation of the arachnoid membrane, and that the lesion of intellect was not so much the effect of local disease of the brain as the result of its complication with an arachnitis engaging the whole periphery of the organ. In the next place, they explain the fact of a *general* affection of the brain, arising from *local* disease, as depending in most cases on the pressure which the tumefied state of the diseased portion necessarily makes on the sound hemisphere; and they state that this pressure must be very considerable, as the brain, being confined within a bony cavity, has no power of expanding itself. Now it is a most interesting fact, in support of this view, that in a great number of the cases of loss of brain, with preservation of intellect *all through the case*, an extensive opening existed in the bones of the skull, so as to permit of expansion in the diseased hemisphere, and prevent the pressure being exercised on the opposite one. This point appears to be borne out by the result of Mr. O'Halloran's cases, and by many other examples. Lastly, in every acute case of local inflammation of the brain, two causes having a tendency to produce symptoms exist. One of these is the local disease which gives rise to those phenomena of motion and sensation which we observe on the opposite side of the body; the other is the determination of blood to *the whole brain*, the result of the irritation of that disease.—'*Ubi stimulus ibi humorum affluxus*.'"

The arguments of the anti-phrenologists, founded on severe injuries of the brain, without corresponding injury to mental manifestations, are delusive. The instances in the first place, are very rare, and only prove exceptions to the general rule. We sometimes see the stomach a mass of cancer, and yet with the power of digestion. Who would argue from this that the stomach is not the organ of digestion? The liver will sometimes be burrowed with abscesses, and yet the gall-bladder will be found distended with apparently healthy bile. Would any one infer that the liver is not the secreting organ of bile? The brain is a double organ, and, though the fusion at the median line is produced by development, yet the symmetrical halves preserve, to a certain degree, their individuality.

"But besides confirming the doctrine, that the brain is the organ of thought, there are innumerable facts drawn from pathology, which have a tendency to prove that particular parts of the brain are the organs of peculiar phenomena. We see an injury of one part of the brain, accompanied by a train of symptoms indicating some peculiar lesion of mind; we see an affection of another part attended by a different class of phenomena. Here pathology, the science which phrenologists reject and despise, goes to establish the ground-work of their doctrines, that the brain consists of a congeries of parts, having each a separate and distinct function. We find, for instance, that disease of one portion of the brain affects the intellect, of another, the generative organs, of a third, the muscular system. What does this prove but that the brain is not a simple organ, but composed of a congeries of parts, each of which governs a different part of the system, or ministers to a peculiar purpose. Now what is this, but what the phrenologists themselves wish to prove?

Further, the professors of phrenology have placed all their organs on the surface of the brain, and for this they have been loudly censured. Phrenology, it is urged, knows, or professes to know, nothing about the central parts of the brain, which must be equally important with the superficial, and have confined their investigations to the surface alone. Now it is a curious fact, that the pathology, which they deny, in this instance furnishes the best reply to this objection. I mentioned at my last lecture, that if we examine the symptom of delirium, we find that it characterises the inflammation of the periphery, and is commonly wanting in that of the deep-seated portions. In other words, mental alienation is the characteristic of the disease of that portion of the brain, where the phrenologists have placed the intellectual organs. Here is a strong fact in favour of the doctrines of phrenology, derived from that science, which the mere phrenologist throws overboard and despises. Again, according to the researches of some celebrated French pathologists, there are a number of facts to shew that there is a remarkable difference between the symptoms of arachnitis of the convexity and of the base of the brain. This conclusion, which after a most careful series of investigations was adopted by them, is borne out by the results of my experience, and appears to me to be established on the basis of truth. They have discovered that arachnitis of the convexity of the brain is a disease characterised by prominent and violent symptoms, early and marked delirium, intense pain, watchfulness, and irritability. We have first delirium, pain, and sleeplessness, and then coma. But in arachnitis of the base of the brain, the symptoms are of a more latent and insidious character, there is some pain, and the coma is profound, but there is often no delirium. What an important fact for the supporters of phrenology is this, and how strikingly does it prove their absurdity in rejecting the lights derived from pathology! Here we find the remarkable fact, that inflammation of the arachnoid, investing the base of the brain to which the phrenologists attach comparatively no importance, is commonly unattended with any lesion of the intellectual powers, while the same inflammation on the convexity is almost constantly accompanied by symptoms of distinct mental alienation.

It is objected to the phrenologists, that they know little or nothing of the central parts of the brain, that though these parts may be fairly considered to be of as much importance as any others, still they do not admit them to be organs of intellect. Now, what does pathology teach on this subject? It shews that we may have most extensive local disease of the central parts of the brain, that we may have inflammation, suppuration, abscess, and apoplexy, without the slightest trace of delirium. Indeed there can be no doubt that the central portions of the brain have functions very different from those on the surface. They appear more connected with another function of animal life, muscular motion and sensation. Then let us examine the phenomena of old age. Every one is familiar with the fact, that when a man arrives at an extreme age, he generally experiences a marked decay of intellectual power, and falls into a state of second childhood. Does pathology throw any light upon this circumstance? It does. From a series of ingenious and accurate investigations, conducted by two continental pathologists, Cauzevielh and Desmoulins, it has been found that a kind of atrophy of the brain takes place in very old persons. According to the researches of Desmoulins it appears, that, in persons who have passed the age of seventy, the specific gravity of the brain becomes from a twentieth to a fifteenth less than that of the adult. It has also been proved that this atrophy of the brain is connected with old age, and not, as it might be thought, with general emaciation of the body; for in cases of chronic emaciation from disease in adults, the brain is the last part which is found to atrophy, and it has been suggested that this may explain the continuance of mental powers during the ravages of chronic disease; and also the nervous irritability of patients after acute diseases, in which emaciation has taken place.

I might bring forward many other facts to shew that phrenology is indebted to pathology for some of the strongest arguments in its favour, and I think that those phrenologists who neglect its study, or deny its applicability, are doing a serious injury to the doctrines they seek to establish. The misfortune is that very few medical men have turned their attention to the subject, and that with few exceptions, its supporters and teachers have been persons possessing scarcely any physiological, and no pathological knowledge. Phrenology will never be established as a science until it gets into the hands of scientific medical men, who, to a profound knowledge of physiology, have added all the light derived from pathological research. To give you an instance of the mode of reasoning of the non-medical phrenologists. In their drawing-room exhibitions, they appeal with triumph to the different forms of the skull in the carnivorous and graminiverous animals with respect to the development of destructiveness; and all are horrified at the bump on the tiger's skull. But, as Sir H. Davy well observes, this very protuberance is a part of the general apparatus of the jaw, which requires a more powerful insertion for its muscles in all beasts of prey. Phrenology, as generally taught, may answer well for the class of dillettantis and blue stockings, or for the purposes of humbug and flattery, but its parent was anatomy, its nurse physiology, and its perfection must be sought for in medicine. The mass of inconsequential reasoning, of special pleading, and of '*false facts*,' with which its professors had encumbered it, must be swept away, and we shall then, I have no doubt, recognise it as the greatest discovery in the science of the moral and physical nature of man that has ever been made. I feel happy, however, in thinking that of late the science has been taken up on its true grounds in Paris, London, and Dublin. Vimont's splendid work on Comparative Phrenology will form an era in the science. In London, Dr. Elliotson has directed the energies of his powerful mind to the subject, and in Dublin we have a Phrenological Society, of which Dr. Marsh is the president, and my colleague, Dr. Evanson, the secretary, and under such auspices much is to be expected."

We cannot follow Dr. Stokes through his extensive lectures on the subject of encephalitis; and we have introduced these extracts and observations, chiefly on account of their bearing on phrenology. Dr. S.'s lectures are calculated to repress the prurient imaginings of the phrenologists, and to lessen the inordinate scepticism of the opposers of the new science. We think, indeed, that Dr. Stokes has taken the view of phrenology which every observant physician, and accurate pathologist will come to at last.

---

### MIDDLESEX HOSPITAL.

### CASES OF POISONING BY BARYTES, AND THE MINERAL ACIDS.*

Our active contemporary, the Medical Gazette, has published three cases, related by Dr. Wilson at the last Conversazione for the present season, held at the College of Physicians. The

* Medical Gazette, July 5th, 1834.

cases in question are instances of poisoning by carbonate of barytes—by nitric, and by sulphuric acid. The first and the last were not fatal, but the second unfortunately was so.

1. *A Case of Poisoning by Carbonate of Barytes.*

A young woman who had fasted for twenty-four hours previously, and who seems to have laboured under some weighty moral depression, half filled a tea-cup with carbonate of barytes, and then filled up the cup with water. She swallowed the whole, in which she discovered no particular taste. Soon afterwards, medicine which occasioned vomiting was given to her.

"On her way to the Middlesex Hospital in the evening, two hours after the event, she found, for the first time, dimness of vision, succeeded by double vision, ringing in the ears, pain in the head, and throbbing in the temples, a sensation of distention, and weight at the epigastrium . she said she felt as if blown up with wind, and complained of palpitations.

When in bed, she first complained of pain in the legs and knees, and cramps in the calves. She vomited twice a fluid like chalk and water, which formed a deposit. Her skin was hot and dry; her face flushed; pulse 80, full and hard. Repeated doses of sulphate of magnesia were given to her.

During the night she had fifteen evacuations; had no sleep, from pain in the head and epigastrium, and ringing in the ears.

The next day she had a hot skin, with profuse perspiration, and slight pain about the pharynx. Her tongue was covered with a white fur, and moist.

A day or two after, the cramps became more severe in all the extremities with a sense of weight, and soreness when touched.

These symptoms, slightly modified, lasted a long time: those which persisted the longest, and which still exist, are severe pains in the head, pain in the left side and epigastrium, great and long-continued palpitations. There has been much difficulty in persuading her to take any sustenance."

She recovered slowly, and left the hospital in the latter end of June.

Orfila has concluded that pure barytes, or the carbonate, produces death by acting on the nervous system, and that it corrodes the parts it is brought in contact with. Mr. Brodie believes, from experiments with the hydrochlorate of barytes, that death is occasioned by its action on the brain and on the heart. In the case related by Dr. Wilson, the nervous and the circulating systems were disturbed; but the fortunate issue prevented the discovery of the actual lesions, if such there were, which the poison occasioned in the stomach.

The appropriate antidote, or rather the most useful remedy, in poisoning by barytes, is thought to be the sulphate of magnesia. This is founded on the fact, that sulphate of barytes is readily formed, and constitutes an insoluble and inactive compound.

2. *Case of Poisoning by Nitric Acid.*

This case, we perceive, is one which has been previously reported by Mr. Arnott, and was noticed in the 38th Number of this Journal. Perhaps it may be thought that the surgeon and physician have looked upon the fact through different glasses—that the medical features have arrested the attention and engaged the affections of the one, whilst the surgical beauties have subdued the imagination of the other. Yet we see so small an amount of variation in the respective descriptions of the surgeon and physician, that we willingly refer to the narration of the former.

3. *Case of Poisoning by Sulphuric Acid.*

A young woman swallowed some oil of vitriol, and immediately afterwards was constrained to take magnesia. She was then taken to the Middlesex Hospital. She laboured under a constant desire to vomit, and what she threw up was of a dark brown colour. Abbreviation of the following circumstances would be useless.

"Two hours after, her lips began to swell; the pain in the throat and stomach increased, with a violent burning sensation. She had a restless night, with frequent vomiting, and a sensation as if she were going to be choaked. Her tongue was covered with a white dense fur, with traces of dark veins, like those of the leaf of a tree; but in vomiting, something like skin was said to have been brought up; since which the tongue has been red. There were scabs forming about the lips, hands, and arms, where the acid had touched them.

During the first four days she was bled to upwards of thirty ounces, and had about 100 leeches applied to the throat; and she took magnesia and linseed-tea, &c.; after which, the abdominal pains became much less, the vomiting less frequent, and she could swallow with less difficulty. Her voice was pretty distinct, with a soft and regular pulse, and a moist and cool skin; but she had two or three shivering-fits, followed by what she called a cold pain felt about the navel.

Two days after, the cough became more troublesome, with constant irritation in the throat, which continued for some time; when, during a violent fit of vomiting and coughing, she brought up a large piece of sloughy membrane, which was found to consist of the inner coats of the œsophagus, much thickened, and very firm in texture; its length was eight or nine inches, and its width that of the œsophagus, being pervious throughout its whole extent. The inside was quite smooth, the exterior ragged; so that the entire mucous membrane, with perhaps some of the circular fibres of the muscular coat, may both be found in the ejected tube which has been preserved, and is now placed upon the table. She experienced great pain in the night after."

The patient had not quitted the hospital in the early part of July, the date of the report. She had been within the walls for six months, and was better during the two last than she had been in the first four. Yet she was rarely able to swallow any thing but fluids, and on the days when she suffered most, she vomited a pint or two of mucous liquid. She had wasted greatly, but her spirits were good.

It would be interesting to be informed of the termination of this case, which appears to have been treated with judgment and decision. The quantity of mucous membrane destroyed in the œsophagus, and, perhaps, some disorganized in the stomach, must render ultimate and perfect recovery difficult, tedious, and uncertain. Should such a recovery take place, it would be curious to observe how the stomach and œsophagus performed their functions, and to ascertain if extensive cicatrization of the latter occasioned any sensible and permanent contraction. Should the patient die, the public would be benefited by a knowledge of the facts disclosed by her dissection.

---

### CITY OF LIMERICK INFIRMARY.

We have received from Mr. Kane, one of the surgeons of the City of Limerick Infirmary, a report of some cases that have occurred under his care in that institution. In an accompanying note, that gentleman alludes to the anxiety we have always shewn for the publication of hospital reports. We are, indeed, most desirous that clinical facts should be closely observed and extensively diffused, and we shall always continue to promote the healthy taste which their study generates. Without further preface, we shall introduce the cases transmitted to us; and we take this opportunity of stating, that we shall feel great pleasure in publishing reports from provincial institutions. All that we require is accuracy in the execution, and brevity in the details.

CASE 1.—*Severe Injury of the Ankle Joint.* Charles Haurahan, 27 years of age, a pilot, was admitted 19th March, 1834. About four hours previously, while steering a vessel past another, near one of the city quays, his right foot got entangled in a coil of rope, and was caught between the ships. On examination of the limb, the foot was found to be twisted inwards, at a right angle with the leg; the inferior extremity of the fibula nearly protruded through the skin, and, were the man held erect, it would be found to be the part nearest to the ground—the skin in front of the joint was slightly contused. By flexing the limb, and making extension and counter-extension, reduction was effected with some difficulty. During the reduction, crepitation was very evident. The limb was placed in the usual position in splints, with bandages, &c.—cold lotions ordered.

On the following day, not feeling much pain in the limb, and anxious to return home, this patient got out of bed, and attempted to walk about the ward; when obliged by the nurse to return to bed, he continued to toss the limb about on this and the following day, which brought on inflammation so violent, as to resist all attempts to subdue it, and which terminated in ulceration and sloughing of the integuments at either side, with profuse purulent discharge from the joint. Hectic symptoms soon set in—the pulse became frequent and small—he had diarrhœa and night-sweats—became much emaciated — appetite impaired, although quinine and other tonics were given. At length it was agreed, in consultation, that amputation afforded the man the only chance of saving his life; he, however, did not consent until the 8th of April, when, assisted by Surgeons Thwaites and Franklin, I removed the limb, at the usual distance from the knee—very little blood was lost; four arteries were tied. The stump was dressed on the 5th day, when it looked healthy, and apparently united, except where the ligatures came out.

April 17th. The condition of the patient is much improved since the amputation—the hectic symptoms have disappeared—the centre of the wound, however, this day, shews some tendency to slough; it was accordingly dressed with powdered bark and lint. None of the ligatures have as yet come away.

April 20th. At 11 o'clock, p.m. hæmorrhage suddenly took place from the stump; and, before the tourniquet was secured, about a pint of blood was lost, which weakened and agitated the patient very much. On my arrival, I removed the dressings and slackened the tourniquet, when a smart jet of blood came, apparently, from the anterior tibial artery. I made three or four attempts to secure the bleeding artery with the tenaculum, but the soft parts giving way, I could not succeed; however, on again slackening the tourniquet, the blood nearly ceased flowing. I then placed a dossil of lint on the bleeding orifice, and filled up the wound with dry lint, and applied compresses and bandages over all.

There was no return of the hæmorrhage—the wound gradually filled up, and was cicatrized by the latter end of May.

On examining the amputated leg, it appeared that the fibula was *unbroken*—the external lateral ligaments of the ankle-joint were torn from the fibula—the malleolar process of the tibia was broken—and the internal part of the articulating surface of the astragalus was broken off, and lay loose in the joint — the synovial membrane was thickened and pulpy, and the joint was bathed in pus.

Case 2.—*Lithotomy.*

John Minahan, aged 12 years, of a pale and sickly appearance, and with a tumid abdomen, was admitted to hospital 22d April, 1834. Has had symptoms of stone in the bladder for upwards of six years, from which he suffered extremely. He was sounded on presenting himself at the hospital, and the presence of the stone ascertained.

He was ordered to be kept quiet in bed—to get half an ounce of castor oil, and flax-seed tea for drink.

April 26th. The existence of the calculus was confirmed by the sound; the boy has been kept on low diet, and repeatedly purged. In consultation, the operation of lithotomy was determined on, and the consent of the boy's parents obtained.

April 28th. A purgative having been administered at 6 o'clock this morning, and the escape of urine prevented by a ligature on the penis, the operation was proceeded on at half past one o'clock, p. m,, assisted by Surgeons Wilkinson and Franklin, and in presence of other medical gentlemen. The usual lateral operation was performed by the common scalpel alone, and the stone extracted, weighing nine drachms and one scruple (troy weight)—very little blood was lost, and the boy sent to bed. The stone consisted of the triple phosphate, at least externally—it was large in proportion to its weight, and was rough at one end, as if it adhered to the bladder. The stone was oval-shaped, nearly 1½ inch long and 1 inch in breadth. I found it necessary, during its extraction, to enlarge the wound with a bistoury, which was passed along the forceps for that purpose. 5 o'clock, p. m. some urine and clotted blood came away by the wound;—the boy does not complain of pain.

11 o'clock, p. m. There is some tenderness on pressure of the hypogastric region, for which 12 leeches were applied, and the abdomen to be fomented. Half an ounce of castor oil to be given at 3 o'clock in the morning.

April 29th. Bowels freed by the oil—feels tolerably easy—pulse quick—tongue whitish—much thirst.

8 o'clock. Feverish symptoms, rather on the increase, and some tenderness of the hypogastric region. He was ordered leeches, warm bath, stupes, and calomel, gr. iij. Pulv. antimonial. gr. iij.

30th. Bowels have been frequently freed by the medicine, has some tenderness and fulness of the abdomen—leeches and stupes to be separated.

9½, p. m. Pulse much quickened—abdomen tender to the touch—countenance shrunk and cold—nausea and slight vomiting—hiccup occasionally, and general uneasiness. Leeches to be applied over the abdomen, a warm-bath subsequently—hot bran poultices over the abdomen, and to have the following draught in whey. ℞. *Aquæ ammoniæ acetat.* ℥ss. *Vini antimonial.* gtts. x. *Tinct. opii,* gtts. xx. M.—to be repeated every third hour, omitting the opium.

May 1st. Some improvement since last report—no tenderness of abdomen—bowels have been moved—pulse not so quick, and skin of natural feel—tongue white and rather dry—slept well during the night—the warm bath to be repeated, and draughts as before.

9½, p. m. Continues easy—to get ℥ss. castor oil towards morning.

May 2d. Bowels freed by the oil—tenderness of abdomen has disappeared—skin natural, pulse not so quick—the urine continues to pass through the wound of natural colour. Diaphoretic draughts to be continued, and warm bath in the evening.

May 3d. Tongue clean—no complaint or uneasiness.

9th. Urine continues to flow by the wound.

19th. The urine has for some days passed partly by the wound, but principally through the urethra. The wound is gradually healing, and the boy rapidly recovering good health. He was discharged from hospital cured, May 26th, and continues in good health.

CASE 3. *Compound Fracture of the Patella, and Wound of the Knee-joint.*

Christopher Delany, æt. 32, a fine healthy young man, was admitted into hospital on the 22d April, 1832. On the previous evening he fell from a height on a heap of stones, one of which caused a compound fracture of the patella of the right leg—the joint was penetrated.

On admission, the limb was much swelled and red about the joint, and considerable pain was inflicted on the slightest motion—a splint was placed under the ham, and simple dressing and a bandage applied, with an anodyne draught at night, composed of xxv. gtts. tinct. opii, and xxx. gtts. vini antimonialis. Bowels to be opened by ol. ricini.

The inflammation continued to increase daily, and at length extensive abscesses formed above, below, and in the joint. Amputation was proposed, but not agreed to at the time. Hectic symptoms now made their appearance, and although quinine, wine, and nourishing diet were given, the man was evidently sinking. He now consented to amputation. The operation (circular incision) was performed sixteen days after the accident, (at the patient's desire, by Surgeon Thwaites, under whose care he was admitted,)—a large abscess, which extended nearly up to the great trochanter, was cut into—the muscles were greatly wasted. The wound continued foul and sloughing up to the 5th June, 1832, when, in consequence of the hospital being given up for the use of cholera patients, Delany was tranferred to the County Infirmary, where he continued to improve until, being seized with cholera, he was sent to the cholera hospital of that parish. After his recovery, he was received here again in the month of July, when the stump presented a healthy granulating surface, but quite cone-shaped, and a large ring of bone protruding. About the middle of August the ring of bone came off, and the stump healed perfectly. In a few days he was dischargd cured.

Case 4. *Compound Fracture of the Patella.*

John M'Namara, 35 years of age, was admitted at 11 o'clock, p. m. April 3d, 1834. A short time before he fell from one of the city quays into a dock, the tide being out at the time. A stone or other sharp substance on which he fell caused a compound fracture of the patella of the right leg. There was considerable hæmorrhage, which subsided when the wound was dressed—and cold evaporating lotions ordered. The wound in the integuments was small and not contused.

On the following day there was considerable swelling and pain over the knee-joint, for which symptoms he was largely bled from the arm—leeches and subsequently cold lotions applied—and purgative medicine ordered.

April 5th. The inflammatory appearances have rather subsided since yesterday—leeches were re-applied, and cold lotions continued. This man being in rather comfortable circumstances, having urgent business at home, and feeling the knee nearly free from pain, insisted on his removal; accordingly, on the following day he was carefully carried to a sail-boat, which conveyed him to his house on the river-side, a few miles from the city. I have since heard from the medical attendant of the dispensary, where this man resides, that he recovered in little more than a week, under the applications of cold lotions to the joint, and aperient medicines.

Case 5. *Compound Fracture of the Leg.*

Thomas Hogan, æt. 40, admitted 2d October, 1833. About an hour before his admission he received a compound fracture of the left leg, about three inches above the ankle-joint. Both bones were broken, and to effect their reduction, the wound was enlarged with a scalpel, and a small piece of the protruded tibia sawn off by means of the chain saw. The limb was placed in splints with the eighteen-tailed bandage. The dressings were not removed until the fifth day, when the wound presented a healthy appearance, and partly united. This man was discharged from hospital perfectly cured on the 9th of November, 39 days after the accident.

Case 6. *Compound Fracture of the Leg.*

Michael Connell, a slater, aged 30 years, was admitted April 9th, 1834. Immediately before his admission he fell from a high scaffold, and got a compound fracture of the leg, about its middle. A considerable portion of the tibia protruded, to reduce which it was necessary to enlarge the wound in the skin. The limb was placed in splints, &c., and cold evaporating lotions ordered. The dressings were removed on the fourth day, and the wound found partly united. This case proceeded favourably—there was exfoliation of a small piece of the tibia—and the man was discharged cured June 2d, 54 days after the accident.

Case 7. *Compound Fracture of the Orbit.*

The following case affords an instance of great tenacity of life in an old person.

Joan M'Namara, 80 years of age, was admitted to hospital at 10 o'clock, p. m. January 18th, 1834. About an hour before her admission she was looking at some persons fighting, and throwing stones at each other, one of which struck her on the upper part of the face, on the right side. The soft parts were severely lacerated, and the bones forming the outer wall and floor of the orbit were broken in several pieces. The finger could be passed as deep as the entrance of the optic nerve into the orbit, and backwards into the temporal fossa. The pupil of the right eye was much dilated, but sensible to strong light. There was considerable ecchymosis of the lids. Several pieces of broken bone were removed, and the wound lightly dressed, and wine and water ordered until the heat of the surface was restored.

Jan. 19th. She passed a restless night until 3 o'clock, a. m., when her bowels were moved, and she vomited once. Since that time she lay in a kind of drowsy slumber—pulse 70, rather hard.

Jan. 23d. In consequence of the edges of the wound shewing a tendency to gangrene, the mild antiphlogistic treatment hitherto pursued was discontinued, and bark and full diet ordered, and stimulating dressings to the wound. The wound soon put on a healthy appearance, florid granulations sprang up with healthy suppuration—small pieces of bone occasionally came away in the dressings. A tonic plan of treatment with some change occasionally, was pursued for some time—the suppuration continued healthy, and the wound looked well—she had low delirium occasionally, at length bed-sores formed—her stools began to pass involuntarily—the granulations now became glassy, and she sank exhausted on the 15th March, 57 days after the receipt of the injury.

During the progress of the case, a fracture was found in that part of the temporal bone forming the base of the skull, through which healthy suppuration flowed at each dressing—the eyeball became shrivelled—of course sight was destroyed—and granulations appeared to shoot from the sclerotic coat of the eye externally. An inquest was held on the body. I happened not to be in town on that day, and the attending surgeon did not think it necessary to open the head, otherwise I would have probably given the details of the case fully, with the result of the post-mortem examination.

---

### Richmond Surgical Hospital.

#### Dr. O'Beirne on Hydrocele of the Neck.

A short notice of this affection will be found at page 513 of the present number of this Journal. A case is there related for the purpose of displaying M. Dupuytren's treatment, and his ideas upon the nature of the malady. Reference is also made to the original description of M. Maunoir, and his use and recommendation of the seton.

Dr. O'Beirne, with whose activity, zeal, and talents, our readers, we are sure, must be familiar, has enriched the pages of our contemporary of Dublin with a lengthened and practical memoir upon this disease. He enters into the history of the opinions which have been expressed, with reference to its nature and the best method of cure, and he does ample justice to the claims and the judgment of M. Maunoir.—With all this, however, we must dispense, and content ourselves with stating the facts and the opinions of Dr. O'Beirne.

We may state that the disease has been frequently confounded with ordinary bronchocele. It actually consists of several serous cysts, which, commencing, of small size, at some point of the side of the neck, gradually increase to such dimensions as to occupy the whole front, and one side of the neck, and seriously impede respiration, deglutition, and speech. The tumor, when established, conveys to the touch a distinct sense of fluctuation, and contains a fluid of either a limpid, a reddish, or a dark coffee colour, and coagulable by heat. In the great majority of cases, it exists independently of any enlargement of the thyroid gland; in one of Dr. Maunoir's cases it was situated behind the angle of the lower jaw, at a distance from the gland altogether. But sometimes the latter is implicated in the disease, and, in the second case related by M. Maunoir, it was enlarged and indurated, and formed one-eighth of the whole tumor.

The cyst, according to M. Maunoir, is usually thick; too much so to permit a cure by the operation of injection. Incision of the cyst, the practice recommended by Dupuytren, and extirpation of part or all of it, he equally considers as too serious and severe. In fact, his practice is to evacuate by puncture the contents of the tumor, and then to pass a seton through its longest diameter. M. Maunoir himself has related four cases, which are severally quoted, though abridged by our author. Dr. O'Beirne details three, which he has personally witnessed, and to these alone we shall now direct attention.

" *Case* 1. Stephen Cassidy, aged 60, of a very wizened, weather-beaten appearance, and residing at Meath-hill, county of Meath, admitted into the Richmond Surgical Hospital, under my care, on the 25th of June, 1831. This man has a very large tumour, which occupies the whole of the front and left side of the neck. At its upper part also, it extends into the left side of the neck, and thence passes obliquely downwards to the left sterno-clavicular articulation, at which point it terminates in a rounded projection, and then sweeps upwards and along the left clavicle to within two inches of the left acromion. The whole of the tumour, particularly that part of it which covers the thyroid gland, is remarkably prominent; gives a perfectly distinct sense of fluctuation, and is quite free from any appearance or feel of pulsation. Its integuments are of a natural colour, and so thinned as to be almost diaphanous; and numerous small veins are seen ramifying beneath the distended skin; but on examining the swelling, in the ordinary way, by transmitted light, it is not found to be transparent at any point. He complains of no difficulty in breathing or swallowing, or of any inconvenience whatever, excepting that arising from the great size and unsightliness of the tumour. He states that the disease commenced about twelve years ago, by a very small, moveable swelling in the centre of the triangular space above the acromial third of the left clavicle; and that this lump had gradually, and, without the least pain, increased to its present size. He is very unwilling to allow the tumour to be opened, and assigns as a reason that a medical gentleman had cautioned him against ever permitting it to be opened, as the consequence would assuredly be instant death by hæmorrhage.

Having, with considerable difficulty, and after a delay of five days, succeeded in removing his fears on this account, I resolved, as this was the first case of the kind that I had seen, on merely making an exploratory puncture into the most depending part of the tumour, which was that corresponding to the left sterno-clavicular articulation. I proceeded thus: a transverse fold of the skin covering this part being raised, it was divided by the shoulder of a lancet; when immediately the sac, very thin, and covered with numerous small veins and arteries, protruded through the incision. The point of the lancet was then passed beyond its shoulders into the protruded sac, and a large quantity of reddish serum discharged. At first, the stream appeared so very red that, fearing it to be of pure blood, it was closely examined, and found to consist of two currents, one serous, and the other very slender, and evidently proceeding from a few small arteries on the outer surface of the sac, which had been divided by the lancet. It was observed also, that almost from the instant that this fluid began to pass off, the tumour began to pulsate, but much more strongly above the left clavicle, than at any other part. In a few minutes, the whole of the tumour was evacuated, and all unusual pulsation ceased. The thyroid gland could now be readily felt, and, after careful examination was found in a perfectly natural and healthy state. Successive layers of lint, steeped in cold water, were then laid along the whole of the left side of the neck, and over these a wet calico roller was applied, so as to exert a moderate degree of pressure. A similar fluid continued to be secreted and discharged by the sac, the dressings became thoroughly soaked, of a reddish colour, and so tightened as to be distressing and require their removal. This discharge continued to flow for three days, wetting, each day, a considerable quantity of old linen, yet without appearing to produce the least debility. On the fourth day it ceased, the opening in the sac and that in the skin having healed; and on the following day, the tumour regained its former size and general appearance.

It was now my intention to again puncture the tumour, and to pass a seton through it; but the patient obstinately persisted in refusing to submit to the second operation, on the plea of an urgent necessity to go home. He was discharged on the 10th of July, faithfully promising, however, to return in a few weeks. Since that time, I have neither seen nor heard of him."

*Case* 2. The patient was a female, aged 60. The tumor had existed for thirteen years, but its growth had been rapid for the last two months. It occupied the front and nearly the whole of the left side of the neck. On its characters, the full description of the other case will preclude the necessity of our saying more, than that the sense of fluctuation was distinct, but of such a nature as to convey the impression of the fluid being contained in a number of distinct cysts. The patient had been admitted into the Anglesey Hospital, under the immediate care of Mr. Hayden. The following operation was performed by that gentleman, assisted by Dr. O'Beirne.

" The skin covering the highest point of the tumour being pinched into a transverse fold, this fold was divided so as to leave a longitudinal wound about an inch long. Some scattered fibres of the platysma were next divided, until the sac came fairly into view. The sac was then freely punctured with a lancet, and a quantity of dark, coffee-coloured fluid discharged. While this fluid was escaping, a blunt probe, armed with a skein of silk, was passed into the opening, and its point made prominent at the most depending part of the tumour. Incisions were then made at this point through the skin and into the sac; the probe was withdrawn, and the silk left in the usual manner of passing a seton. The tumour being now completely emptied, the thyroid gland was carefully examined, and found quite free from enlargement, hardness, or any other morbid condition perceptible to the eye or touch. On examining the sac also, at its upper part, and separating it from the parts beneath, which was easily effected, another but much smaller cyst was clearly seen and felt at a considerable depth, and situated so directly over the carotids, that it was not considered safe to puncture it."

The subsequent events and the treatment adopted require no especial notice. The seton was withdrawn on the 25th June, after a residence of about a month. The patient was discharged cured on the 10th of July, some cervical glands being then in a condition of chronic enlargement.

The patient was again admitted into hospital, on the 17th of September following, with a small fluctuating tumor, situated about one inch above the left clavicle, and crossed obliquely, at its centre, by the external jugular vein. Mr. Hayden, assisted by Drs. O'Beirne and Ireland, operated upon it in the same way that he had upon the former, and gave exit to a comparatively small quantity of the same kind of fluid. Except slight constitutional disturbance, and accumulation at one or two points which required to be opened by a lancet, nothing remarkable occurred in the progress or treatment of the case; and the woman recovered, with merely a small indurated elevation marking the seat of the second tumor.

We need not detail the particulars of the third case. It is sufficient to observe, that the tumor was seated in the left parotid region—that it presented much the appearance of fungus hæmatodes—that it was punctured on several occasions—that at first, clear reddish fluid issued, and afterwards a liquid resembling coffee-grounds, and containing some hydatids—that some inflammatory action occurred in the tumor—and that, finally, the patient left the hospital of his own accord, the tumor having at that time given way, and discharging a good deal.

Dr. O'Beirne defends, with cordiality and vigour, the nomenclature and the views of Dr. Maunoir. We shall content ourselves with citing the concluding observations in the essay. They refer to an improvement, suggested by the author, in the manner of introducing the seton.

" Circumstances have convinced me that the operation which I have described, may be considerably improved. In assisting Mr. Hayden, I observed that when the point of the probe was made prominent at the most depending part of the tumour, and he attempted to cut upon the end of the instrument; the integuments glided from side to side, and so effectually evaded the shoulder of the lancet, that it was necessary to make several attempts before the incision could be completed. This was evidently owing to the fluid contents escaping at the upper opening, and rendering the tumour so flaccid as to enable the integuments to glide freely over the sac. In order to obviate this defect, which causes unnecessary pain to the patient, and an awkward kind of embarrassment to the surgeon, it will only be necessary to expose the sac, previous to puncturing it, at the upper and lower extremities of the tumour, by raising and afterwards dividing a transverse fold of the integuments at each of these points. By proceeding in this way, the sac alone opposes the passage of the probe through the lower opening, and no difficulty will be found in cutting into it, so as to allow the instrument to pass, and the seton to be introduced.

After the seton has been passed, I am now disposed to alter my opinion respecting the applications of either a roller or water dressings. I believe that applying a simple dressing, or a light, warm, emollient poultice, would be better practice.

To conclude.—It is somewhat remarkable that, out of ten cases which have been related by Heister, Maunoir, Lawrence, and myself, and in which the side affected is mentioned, nine have occurred on the left side of the neck. The late M. Delpech, whose untimely and awful fate we must all deplore, does not, if Mr. Lawrence's extracts may be trusted, mention the particular side on which either of his two cases occurred. I have seen the first, but have not been able to procure the second, volume of this very distinguished French surgeon's work."

The paper requires no further comment, than the recommendation to peruse its facts, and the expression of thanks to the able author, for directing the attention of the profession, in this country, to the nature and the treatment of cysts, containing aqueous fluid, in the neck.

---

### Meath Hospital.

### Observations on the Treatment of various Diseases. By Robert J. Graves, M.D.

This is not altogether a clinical lecture, and yet it has more of that character than of any other. It is a running expression of Dr. Graves' experience, displaying the familiar ease and the ready illustration of the clinical chair. It occupies thirty pages of our contemporary, but the cream may be advantageously skimmed from that extensive surface.

The subjects on which Dr. Graves discourses are, 1, On effusion of air within the chest in inflammation of the lungs—2, Bruit de soufflet of the heart and throbbing of the chest in pneumonia—3, Symmetrical erysipelas—4, On the best method of administering calomel in acute inflammations—5, Spontaneous cure of chronic ascites—6, Diffuse inflammation terminating fatally in consequence of effusion within the chest—7, Loss of the sense of smelling—8, Carbonate of ammonia in the urine—9, On albuminous urine in dropsy—10, On diabetes insipidus.

I. *On Effusion of Air within the Chest, in Inflammation of the Lungs.*

Dr. Graves details a case of what he considers, and of what, indeed, appears to have been, simple pneumo-thorax, —that is, of pneumo-thorax unconnected with tubercles in the lungs. The particulars of the case may be briefly stated.

A gentleman, æt. 40, of robust constitution and make, caught cold, and was attacked with cough, pain in the right side, bloody expectoration, and, in short, the usual symptoms of very intense pneumonia, commencing in the inferior portion of the right lung, but advancing rapidly upwards, until the whole of that lung was engaged in the disease. As the inflammation extended, the inferior portion of the lung became engorged with blood, and totally impervious to the air, and consequently the part of the chest corresponding to it every where yielded a dull sound on percussion, while the superior part of the right side was as sonorous, when percussed, as the left or healthy side of the chest. Such was the state of things on the third day of the disease. On the morning of the fourth day, a remarkable change was found to have occurred in the course of the night; anteriorly, the dulness of the lower portion of the affected lung still continued, and, indeed, could not be greater, but from a little below the right mamma, as far up as the clavicle, which region, at the preceding visit, was naturally sonorous, the chest yielded a preternaturally clear and hollow sound. Where the sound was thus distinct, no respitory murmur could be heard. The opposite lung presented well-marked puerile respiration, whilst the sound upon percussion was naturally clear, and, of course, less sonorous than in the upper portion of the right side.

From all these circumstances, Dr. Graves and Dr. Marsh concluded, as they necessarily must, that air was effused into the cavity of the pleura, while the lung was, in consequence, compressed. The previous health and the excellent constitution of the patient, rendered it unlikely that the pneumo-thorax was occasioned by the presence of tubercles in the lung. They were, therefore, compelled to come to the conclusion, that the air was secreted by the inflamed pleura. The progress of the case supported the opinion.

"At our next visit, in about sixteen hours after, we found the whole region, that had been preternaturally clear on percussion, now dull as possible, and presenting a very obscure respiratory murmur, mixed with some crepitus. The crepitus was evidently close to the ear, if I may use that expression, and we now felt no doubt that the air so suddenly effused, had been as suddenly absorbed, and its place occupied by the inflamed and engorged lung. In the course of four or five days, under proper treatment, this dulness began to diminish, and nearly disappeared in a few days more, during which time the respiratory murmur proportionably increased, and the gentleman afterwards rapidly recovered."

Dr. Graves remarks that this, and a case which he has previously published, are fully sufficient to establish the existence of simple pneumo-thorax. We believe that this was recognized, and received into the list of human maladies, before Dr. Graves even dreamt of looking for it. Laennec expressly declares that simple pneumo-thorax sometimes occurs; and we strangely misconceive both him and Dr. Graves, if the latter can lay claim to distinguishing or describing a new variety of the disease. We have witnessed two instances of what seemed this affection. One case was remarkable. A schoolmaster, after exposure to cold, was attacked with all the symptoms of acute inflammation of the left lung and pleura. After a time, the side became prominent, was unnaturally resonant upon percussion, and the respiratory murmur could scarcely be discerned. What little there was seemed distant, and was bronchial. Under the treatment adopted, the resonance slowly diminished, and respiration as slowly returned. The patient ultimately recovered, and is now in the enjoyment of good health. We supposed, and still suppose, that this was an example of pneumo-thorax, unconnected with tubercles and vomicæ. Dissection has not verified conjecture in any of these instances.

II. *Bruit de Soufflet in the Heart and Throbbing of the Chest in Pneumonia.*

"Another phenomenon (says Dr. Graves) observed in the progress of the foregoing case, strongly attracted the attention of Dr. Marsh and myself, bruit de soufflet, of the most distinct and loudest sort, audible not merely in the region of the heart, but over the entire front of the chest. This *bruit did not exist in the subclavian or carotid arteries;* Dr. Marsh, who watched the case with the utmost care, is quite certain that no such sound accompanied the action of the heart in the commencement of the pneumonia; it was not until considerable dulness and disappearance of respiratory murmur over the lower portion of the lung had taken place, that the bruit de soufflet began, increasing in intensity as the inflammation of the right lung spread upwards. This new symptom caused us much uneasiness, and naturally induced the fear that the inflammatory action was not confined to the right lung, but had extended to the heart and great vessels, an occurrence that would have rendered the case almost hopeless. Our fears made us attend to this symptom with the greatest anxiety. For several days it continued without the slightest abatement, but at the period when the stethoscope and general symptoms indicated a notable diminution of the inflammation, then the bruit de soufflet began to diminish in loudness and intensity, and in the course of four days altogether disappeared."

The fact is worth recording; the explanation is uncertain. Throbbing of the chest was another feature of the case in question. It was as strong at the right mamma, and even far above it, as it was directly over the heart itself. Dr. Graves' suppositions on the mode of its production are long, and may be just. The following statements are less liable to doubt. In a case of pneumonia attended by Mr. M. Collis and himself, the action of the heart was very powerful, and a distinct pulsation, corresponding to each ventricular stroke, was perceptible in all the veins of the back of the hand. In a lady, who was attacked with acute peritonitis, venous pulsation was evident to Dr. Ireland, Mr. Crampton, and Dr. Graves.

III. *Symmetrical Erysipelas.*

Dr. Graves relates an instance of erratic cutaneous erysipelas, which travelled at an equal pace, and in a manner exactly similar, upon either side of the median line. The fact is curious, and that is all that can be fairly said of it. We have seen abundance of erysipelas, but we never witnessed an instance of this sort. The practitioner may consume the greater part of his life before he enjoys an opportunity of seeing it; and when that opportunity arrives, it amounts to nothing more than the tickling of sorry curiosity. Two wood-cuts are expended upon it by our author.

Le jeu ne vaut pas la chandelle.

IV. *On the best Mode of administering Calomel in Acute Inflammation.*

Dr. Graves makes some bold and excellent remarks on the exhibition of mercury, in cases of acute inflammation of important organs. He refers, in a highly complimentary manner, to the practice recommended and adopted by the senior Editor of this Journal. Of that practice he approves, and his own would appear to have been founded on it. The practice may be briefly stated.

The patient must take no cold fluids; whatever he drinks must be moderately warm; barley water, without lemon juice, should be preferred; and he should not consume more than three pints of drink in the twenty-four hours, as too much drink disturbs the stomach and bowels, and favours mercurial diarrhœa. Grapes and all fruit must be withheld, a precaution too often entirely neglected, much to the injury of the patient. With these precautions, Dr. G. gives calomel in scruple doses, one daily being usually sufficient, but imminent danger sometimes demanding a second dose, after the expiration of twelve hours. The supplementary remarks, the cautions, and advice of Dr. Graves are judicious.

V. *Spontaneous cure of Chronic Ascites.*

A lady had for *thirteen* years been suffering from a gradual accumulation of fluid in the abdomen. This greatly exceeded in dimensions that of a woman in the ninth month of pregnancy. Yet her health was good, and no operation was attempted. About six months ago, the complaint having been stationary for the previous year, the catamenia, which had constantly been scanty, suddenly became frequent and profuse—a copious discharge of urine succeeded—in less than a week night sweats were established—and, in about a fortnight from the commencement of the diuresis, no vestige of ascites was perceptible. The lady went again into society, slim and attenuated as a purged leech.

VI. *Diffuse Inflammation fatal from Effusion into the Chest.*

There is much to be said, and something to be unwritten on the subject of diffuse inflammation. The paper of Dr. Duncan is confused and unsatisfactory, and much of the error and obscurity that may be found in it are owing to the imperfect information of that day, with respect to the secondary inflammations and deposites. We entertain but a mean opinion of the essay of Mr. Lawrence, on Erysipelas Phlegmonodes. It is neither particular nor comprehensive, and it wears the livery of the rhetorician and the advocate, rather than that of the philosophical searcher after truth.

The cases before us, two in number, are not deficient in practical interest, yet we cannot afford to copy their details. The following are the more important circumstances.

*Case* 1. The patient, a male, aged 22, was admitted into the Meath Hospital, Nov. 11, 1833, with fever of a typhous character, and symptoms chiefly referable to the head. The symptoms had existed for five days. On the seventh day after the commencement of his illness, he was attacked with epistaxis, and much night delirium. On the eleventh day, he became deaf. After this he improved, but trifling bronchitis was established. On the 21st day he was seized with rigors, succeeded by fever, sore throat and dyspnœa. On the next day the latter symptom had increased, and respiration was attended with a croup-like noise; a good deal of swelling existed on the external part of the throat beneath the lower jaw, which parts were scarcely red, but extremely tender to the touch. The same sort of œdema existed internally, occupying the velum, uvula, and tonsils, so as nearly to close the aperture of the fauces. The mucous membrane lining these parts had a transparent appearance, and was but little, if at all, redder than natural. Slight dulness existed in the postero-inferior portions of both lungs, and moist bronchitic râles were distinguished. On the next day all the symptoms seemed relieved. On the next the respiration was more difficult and hurried. On the following day he died.

*Dissection.* The muscles of the neck were pale and soft, and the cellular tissue infiltrated with a yellow sero-gelatinous fluid. The sternum and cartilages of the ribs being raised, the cellular tissue between the anterior mediastinum and the sternum presented the same appearance as that in the neck. Effusion into both cavities of the chest, amounting to two quarts of a sero-sanguinolent fluid, in which floated flocculi of lymph. Lymph deposited on the surface of both lungs; but most evident over the interlobular cellular tissue: it was soft, and only occurred in patches. Cellular tissue of pericardium infiltrated with the same jelly-like substance as noticed on anterior mediastinum. Heart firm and contracted, the pericardium full of turbid serum, containing flocculi of lymph, and at the base of the right ventricle *an ecchymosed spot about the size of a shilling.* The substance of the lungs was crepitant, and no spots of induration could be detected.

On opening the larynx, the chordæ

vocales were found slightly thickened, but otherwise healthy, as were also the parts about the pharynx.

*Case* 2. A female, æt. 50, admitted into the Meath Hospital. She had suffered from pain in the right side for nine days, and for three or four from a painful swelling of the upper part of the same side of the chest and neck; the swelling was diffuse, colourless, and extremely tender, and pitted slightly upon pressure. The respiration was hurried; the lips were rather livid. There was slight delirium. Dyspnœa increased, and two days after her admission she died.

*Dissection.* Infiltration of serum and lymph in the cellular tissue of the neck and thorax—whiteness and softening of the pectoral and sternal muscles—cellular membrane of the anterior mediastinum infiltrated with serum and much lymph—bloody fluid in the pleural cavities, and petechial spots on the serous membrane of the lungs and heart—and softening of the latter were the principal morbid alterations discovered.

The low character of the cellular inflammation in the preceding cases—the late period of its occurrence—and the complication of low inflammation of the pleura and the pericardium are characters worthy of attention. The fact is, that diffuse cellular inflammation, in whatever part of the body it is situated, frequently induces, or at all events is attended with, pleuro-pneumonia or pericarditis. We might almost affirm, that we have witnessed innumerable instances of this description; we have certainly seen many. But we cannot enter on this wide, and, to us, seductive subject. We shall probably revert to it fully at a future time.

VII. *Loss of the Sense of Smelling.*

" I had lately an opportunity of observing a very singular case of the total loss of the sense of smelling, occasioned by exposure to the effects of a very strong and disagreeable odour. Mr. ——, formerly a captain in a yeomanry corps, was attended by Mr. Barker of Britain-street and myself. He was affected with ascites, and in the course of conversation one day, mentioned that in the Irish rebellion of 1798, information was received by the magistrates, that five hundred pikes were concealed in one of the markets of this city, buried at the bottom of a large cess-pool, which was filled with the offscourings of the market and all manner of filth. He proceeded to the place, and superintended the work of emptying out the cesspool, at the bottom of which the concealed arms were found as specified. During this operation he was exposed to most abominable effluvia, and suffered greatly at the time from the stench. Next day he found that he had become entirely insensible to odours, and since that, now a period of thirty-six years, he has remained completely deprived of the sense of smelling. From this it appears, that as exposure to very intense light may produce amaurosis, so exposure to intense odours may produce a corresponding affection of the olfactory nerve."

VIII. *Carbonate of Ammonia in the Urine.*

Dr. Graves had noticed the existence of this salt in a former case, which he published several years ago. The patient, in the present instance, was an athletic man, who, after imprudent exposure to cold, was attacked with anasarca, ascites, and intestinal tympanitis. There were pain and tenderness in the belly, and antiphlogistic treatment was necessary. The urine was now found to contain the carbonate of ammonia in some quantity.

" It was of rather a pale straw colour, contained no albumen, and acted on the vegetable colours as an alkali. It deposited a precipitate consisting of the ammoniaco-magnesian phosphate and phosphate of lime. This remarkable urine was supposed by some who witnessed the violence of its effervescence on the addition of an acid, to owe the formation of its ammoniacal salt to decomposition during its retention in the bladder. But that this was not the source of the carbonate of ammonia, was proved by many circumstances.

It was perfectly limpid when voided, and had not the slightest smell of putrescence, such as exhales from urine even in the commencement of decomposition. Again, when our patient completely emptied the bladder of its contents, and in half an hour afterwards again passed a small quantity of water, this latter was found as copiously loaded with carbonate of ammonia as the former. It necessarily follows, therefore, that the urine, as secreted by the kidneys, contained the carbonate of ammonia which seemed to be a vehicle for excreting those elements which are usually combined so as to form urea, *for in this man's urine, not a trace of urea could be discovered.*

The occasional presence of ammonia in the urine, in the form of the ammoniaco-magnesian phosphate, has been long known to chemists; carbonic acid is of much rarer occurrence indeed, for not more than one or two cases have, I believe, been observed, in which carbonate of lime has been found forming a urinary calculus in the human bladder, although so common in swine and other animals."

The patient died. On examining his body, the kidneys were discovered to be rather enlarged, and somewhat turgid with blood. The bladder was perfectly healthy. The liver was misshapen, round at the edges, smaller than natural, indurated, and composed throughout its whole mass of globular masses, very firm and pale, forming a variety of what is called scirrhous liver.

IX. *Albuminous Urine in Dropsy.*

Dr. Graves is one of those who cannot admit that the presence of albuminous urine in dropsy is conclusive evidence of the existence of structural change in the kidneys. He has seen so many instances of the disappearance of this symptom under proper treatment, that he feels compelled to come to the conclusion, that it may be, and frequently is, produced by functional derangement only of the secreting organ.

We cordially agree with Dr. Graves. Albuminous urine is not unfrequent in cases of disease of the urinary organs, and indeed under circumstances where no disease of those organs can be apprehended. A patient had caries of the tarsal bones, and ulceration of the soft parts of the foot. One day we were tempted, from mere curiosity, to examine the condition of his urine. It was loaded to excess with albumen. The patient was rather broken down in health, but laboured under no apparent affection of the urinary organs. The albuminous condition of the urine continued for some time and gradually disappeared. Another individual had popliteal aneurism, for which the femoral artery was tied. The wound was slow in healing. He was attacked with symptoms indicating the descent of a calculus from the kidney to the bladder. The urine became albuminous. The calculus was voided by the urethra, and proved to be composed of phosphate of lime. In the course of a short time the patient died from an accidental attack of erysipelas. On examining the body, the kidneys, the bladder, and the prostate appeared to be perfectly healthy.

To return to Dr. Graves, he believes, with Frank, that some cases of dropsy may be analogous to diabetes.

"An attentive observation of the different forms under which dropsy presents itself, led me to the following conclusions. When dropsy comes on gradually, is chronic, and unattended by any evidence of being caused by inflammation either of the chest or belly, and where we cannot detect the existence of organic disease either in the thoracic or abdominal cavity, then there is some reason to suspect that the dropsy may be analogous to diabetes. If, in addition to these characters, the urine is found either more copious, or as copious as natural, and especially if it is found to be albuminous, then our suspicions are strengthened, and we are justified in trying the peculiar method of treatment which this variety of dropsy demands, and which consists not in bleeding or leeching, not in purging or exhibiting diuretics, not in mercurializing the system, but in the use of opium and animal food in moderate quantity. Of the success of this treatment in such cases, (but in such only,) we have had several striking instances in the Meath Hospital."

A case is related in illustration, but it does not demand particular notice.

X. *Diabetes Insipidus.*

Our author details two cases of this affection, as contributions towards its history and treatment. Yet those cases are unsatisfactory, for the patients left the hospital too soon to decide the permanency, or otherwise, of their recovery. A remark of Dr. Graves is deserving of attention.

"Although the urine differed so considerably in specific gravity and chemical composition from that of saccharine diabetes, yet did its qualities and quantity become natural, and the general health improve under the use of the remedies found most beneficial in diabetes mellitus, so that whatever distinction may be made between these affections, according to the nature of the urine, they seem nearly identical as to the constitutional symptoms accompanying them, and the mode of treatment to be employed, if I may judge from the three cases referred to."

Dr. Graves observes, with reference to diabetes mellitus, that although an arid state of the skin is generally present, a tendency to perspirations is sometimes displayed. In the case of a gentleman, in whom the disease had existed for a year, and in whom the daily amount of urine was about six pints, of the sp. gr. 1051, and highly loaded with saccharine matter, exhausting night perspirations were complained of. Dr. Graves saw a similar case, in 1820, under the care of the late Dr. Duncan.

Here we close this notice of a lengthened, yet as valuable as lengthened, article. We have taken many opportunities of noticing in terms of high, and of merited commendation, our contemporary of Dublin. It continues to do credit to its immediate conductors, and to those whose contributions occupy and adorn it. We trust that the *principes* of the profession in Ireland will continue to support the only medical periodical of which Ireland can boast. These are times for all to be up and stirring, and we trust that the activity of Irishmen will not continue to be chiefly shewn in religious animosities and the use of the shilelah.

---

MANCHESTER ROYAL INFIRMARY.

CLINICAL LECTURES. By Dr. CARBUTT.*

We have said so much, and discoursed so often on the benefits to be derived from the publication of hospital reports, as well as from the systematic delivery of clinical lectures, that we will not sing again that cuckoo-note. Yet we think we may fairly lay claim to the merit, of having aroused the medical officers of hospitals and infirmaries to consult their own interests and those of science, by diffusing a knowledge of the varied and important facts which present themselves to notice in public institutions. We have doubted, and we still doubt, whether separate and bulky volumes constitute the best mode of publishing clinical lectures and reports. We are sure it is expensive; we believe it is, therefore, inefficient. Many a hospital surgeon or physician is wisely deterred by prudential considerations from the venture—and the work when published is seldom purchased. A well-arranged system of quarterly reports in the pages of a periodical would probably be the only plan likely to enjoy stability or attain success.

We shall liberally draw from Dr. Carbutt's work on various future occasions. On the present, we shall offer a brief sketch of its nature—and direct a hasty glance at its object.

The diseases which occupy the attention of Dr. Carbutt are Gastro-enteritis—peritonitis—jaundice—bronchitis—phthisis—pleurisy—scarlet fever—ague—rheumatism—amenorrhœa

---

* Clinical Lectures in the Manchester Royal Infirmary. By E. CARBUTT, M. D. Octavo, pp. 407. London, 1834.

—feigned disease—chorea—painter's colic—secondary syphilis—dropsy—and diabetes. Some of these affections are honoured with a greater, some with a smaller degree of consideration. The details of one hundred cases are presented, and they occupy the principal portion of the volume. The observations are generally brief, and such as naturally spring from the facts.

The object of the work may be learnt from some remarks contained in a short introduction to the cases. Before we advert more particularly to it, we must seize the opportunity of quoting one passage, intended to urge the medical practitioner to recollect that he is, and must ever continue, a student.

"The progress of science," says Dr. Carbutt, "is so rapid, that if you once consent to give up study, and to satisfy yourselves with the knowledge acquired up to any particular period, you will remain like a ship at anchor when the tide is driving fast, and will have the mortification of seeing every little boat shooting a-head of you, and leaving you further and further behind."

The remark may be trite, yet its truth is such that it cannot be too forcibly impressed on the profession. Examples of such stationary gentlemen as Dr. Carbutt has depicted are too frequent, even in the highest places. The veteran apothecary and the old physician are too often specimens of antiquated ignorance, and respectable imbecility. Disdaining the new-fangled notions of pathology, they hug the good old precepts, and prescribe the good old remedies; and if haply some opinionated "nervous fever" *will* have its way in spite of even the best-concocted juleps, why the patient dies as others died before him, according to the rules and the practices of art. By the way, the nautical metaphor of Dr. Carbutt is not altogether unexceptionable. Had Manchester been near the sea, Dr. Carbutt would probably have found that ships at anchor, "when the tide is driving fast," seldom experience much mortification from seeing little boats shoot *a-head* of them. The tide is usually observed to run in the opposite direction, and little boats, as well as big ones, are commonly very glad to swim with it.

But we wished to exhibit the object of this volume. It is this.

"Clinical lectures, I have said, give you an opportunity of seeing and studying cases, so as to render you in some degree familiar with diseases and treatment. And yet I feel it to be my duty to caution you against becoming what may be called case-practitioners. You must practise from principles and not from cases."

And further on Dr. Carbutt proceeds:—

"Now, I would recommend you to study as many cases as you can, but solely with a view to enable you to form, correct, or strengthen your principles of practice. If you see that either sensibility or the power of muscular motion is lost, you may be assured that the nervous system, in some of its parts, either the brain, the spinal cord, the ganglions, or the nervous filaments are, or have been, oppressed in some manner, or otherwise injured. You must endeavour to discover the cause of this, and then to remove it. Of the means of accomplishing these objects, we shall afterwards have occasion to speak.

Again, from the cases you have witnessed or will witness, you may form this conclusion, that the most likely mode of lessening entonic inflammation in any part of the body, is to lower the action of the heart both in force and frequency; and that the way to do this is to abstract blood from the circulating system. You will, therefore, bleed in acute rheumatism, in inflammation of the brain, of the ear, of the throat, of the larynx and windpipe, of the lungs and the pleura, which last inflammation is called pleurisy, in inflammation of the heart, in peritonitis, in inflammation of the stomach, of the intestines, of the liver, of the spleen, of the kidneys, of the bladder, and in various other inflammations: that is to say, if all the inflammations which I have enumerated, and also those which I have not enumerated, be acute or entonic.

In like manner you will lay it down as a principle that the same abstraction of blood, for the same reason, is the best method of arresting entonic hemorrhage; that is, the vigorous and spontaneous effusion of blood from any of the outlets of the body. You will, therefore, draw blood in entonic bleeding at the nose, entonic spitting of blood, entonic vomiting of blood, entonic bloody urine, entonic uterine hemorrhage, entonic anal hemorrhage.

So, you will find, that in entonic inflammation, and in entonic hemorrhage, except perhaps, that those affections be seated in the inner or mucous membrane of the alimentary canal, an excellent assistant to the abstraction of blood is the administration of purgatives. You will therefore give purgatives proportioned to the age and strength of your patients. In inflammation or hemorrhage of the mucous membrane of the alimentary canal, you must abstain from violent purgatives, for reasons which I shall have frequent opportunities of explaining.

You will also soon discover the principle that the administration of mercury so as slightly to effect the mouth is an almost certain method of overcoming inflammation in all the membranes or textures; except, perhaps, the mucous membranes, for I am not quite certain about them. You will, therefore, administer mercury, in an appropriate manner, in all inflammations of serous membranes, of cellular texture, of muscular texture, of tendinous, and ligamentous texture, of nervous texture, of dermatic texture or skin, and of cartilaginous texture.—That mercury has the same power in inflammations of mucous membranes has been asserted. I do not mean to deny it; but I have some doubts."

It appears to us that the preceding doctrine concerning principles is not so clear as Dr. Carbutt seems to think. We will not turn his position by a joke, nor do we intend to lay stress on the case of the philosophical tailors of Laputa, who, cutting their coats upon abstract principles, were the very worst *fitters* that Gulliver had seen.

But what are principles in medical science? Are they truths derived from some genuine revelation—or abstract expressions, the product of some unerring mental operations? Perhaps a slight analysis of the principles directly stated by our author, may assist us in determining their nature. It is a principle, then, according to the Doctor, that "mercury, administered so as slightly to affect the mouth, is an almost certain method of overcoming inflammation in all the membranes or textures, excepting, perhaps, the mucous membranes." Following out this principle, Dr. Carbutt tells his pupils to administer mercury, in an appropriate manner, in all inflammations of cellular texture—of dermatic texture (that is, skin)—and of cartilaginous texture.

A little consideration will render it apparent, that "principle," in these instances, means nothing more than the expression of the fact; that fact could only be determined by experiments; and those experiments were surely cases. Supposing the principle, then, to be correct, it resolves itself into a simple expression, an algebraic exponent, of many individual cases.

We contend that this erection of "principles" into rules and guides of conduct is pernicious to the understanding of the pupil. It is a seemingly short cut to truth, which frequently leads into a bog. It resembles the adoption of saints and martyrs, as intercessors in the Catholic faith; those saints and martyrs at length usurped the honours of the Deity. We profess ourselves Iconoclasts, and would shiver these symbols of idolatrous adorers.

To revert more closely to the question. We repeat, that a "principle," in physic, implies nothing more than the constant occurrence of a fact—that it must have been established, and can only be maintained, by the actual observation of cases—that the process by which itself was deduced can never be injurious to the minds of students; for, if the principle is strictly accurate, the student should still examine for himself, and learn to test its accuracy by personal investigation. This is the true spirit of induction, the highest exercise and noblest privilege of reason. To erect even truths of demonstration into laws which should not be questioned, and need not be examined, would be to erect a debasing despotism in the intellectual world. What would be thought of the geometrician, who asserted that the sides of an equilateral triangle were equal to each other, yet objected to the careful examination of the problem? The case is analogous to that of the lecturer, who affirms certain laws, which can only be proved or rebutted by the careful observation of cases, yet requires that the pupil should study cases with the conviction, à priori, that those laws are true.

We have put the case in a favourable manner, by supposing that the "principles" are actually true. But if they should be doubtful, or absolutely false, we need scarcely say that the mischief would be seriously aggravated. We have no hesitation in affirming, that some of the principles of Dr. Carbutt *are* of dubious character. He asserts, for instance, that the use of mercury, so as slightly to affect the mouth, "is an almost certain method of overcoming inflammation in all the membranes or textures, except, perhaps, the mucous membranes." And, acting on the principle thus laid down, Dr. Carbutt, as we said before, decidedly tells his pupils to exhibit mercury, in all inflammations of cellular, dermatic, and cartilaginous texture. Erysipelas is inflammation of the skin, and diffuse cellular inflammation is seated, of course, in the cellular texture. We have no hesitation whatever in asserting, that mercury, administered so as slightly to affect the mouth, is always unnecessary, and frequently injurious, in the first affection—and that no good surgeon would, in the great majority of cases, consider it prudent to resort to it in the second. Here, then, is a wide separation of opinions, on what is held up to inexperienced pupils as a fundamental principle.

Our own advice to pupils is this. Be led till you are able to run alone—but not one moment longer. Look on all laws and all principles (we allude to medicine) with considerate distrust—recollect that these are drawn from the observations of others, that Nature is before you, and that you have as good a right to observe as they. We give not this advice to encourage arrogance, but to promote induction. Without that, there is no sound and stable philosophy—no laws that can claim obedience—no generalizations that are safe or certain.

Dr. Carbutt alludes, with a feeling of contempt, to what may be called the mere "case-practitioner." We have seen that he contrasts with this plodding hack his thorough-bred, principle-fed racer. The former is, indeed, a sluggish beast, but the latter is gingered, and got up for show.

The fact is, that the very best men in our profession are what Dr. Carbutt calls "case-practitioners." But case practitioners are not all alike. Some men witness cases—some observe them. The former constitute the mere routinists, men who do not rise above the comprehension and the treatment of symptoms; such are Dr. Carbutt's case-practitioners. The other class to which we have alluded watch the phenomena which cases present—they analyse, combine, compare—and the results are generalizations, or, as Dr. Carbutt terms them, principles, which serve to facilitate the comprehension, and assist the management, of the cases that shall follow. This, however, is a different thing from setting out with laws and principles ready-made, and calculated to control rather than to help.

In succeeding Numbers, we shall analyse or notice, as circumstances may require, the cases and the observations contained in the volume. In the interim, we recommend our brethren to peruse it.

